# Diverse Hormone Response Networks in 41 Independent *Drosophila* Cell Lines

**DOI:** 10.1534/g3.115.023366

**Published:** 2016-01-12

**Authors:** Marcus Stoiber, Susan Celniker, Lucy Cherbas, Ben Brown, Peter Cherbas

**Affiliations:** * Department of Biostatistics, University of California Berkeley, California 94720; § Department of Statistics, University of California Berkeley, California 94720; † Department of Genome Dynamics, Lawrence Berkeley National Laboratory, California 94720; ‡ Department of Biology, Indiana University, Bloomington, Indiana 47405

**Keywords:** ecdysone, network biology, transcription, RNA-seq, bioinformatics

## Abstract

Steroid hormones induce cascades of gene activation and repression with transformative effects on cell fate . Steroid transduction plays a major role in the development and physiology of nearly all metazoan species, and in the progression of the most common forms of cancer. Despite the paramount importance of steroids in developmental and translational biology, a complete map of transcriptional response has not been developed for any hormone . In the case of 20-hydroxyecdysone (ecdysone) in *Drosophila melanogaster*, these trajectories range from apoptosis to immortalization. We mapped the ecdysone transduction network in a cohort of 41 cell lines, the largest such atlas yet assembled. We found that the early transcriptional response mirrors the distinctiveness of physiological origins: genes respond in restricted patterns, conditional on the expression levels of dozens of transcription factors. Only a small cohort of genes is constitutively modulated independent of initial cell state. Ecdysone-responsive genes tend to organize into directional same-stranded units, with consecutive genes induced from the same strand. Here, we identify half of the ecdysone receptor heterodimer as the primary rate-limiting step in the response, and find that initial receptor isoform levels modulate the activated cohort of target transcription factors. This atlas of steroid response reveals organizing principles of gene regulation by a model type II nuclear receptor and lays the foundation for comprehensive and predictive understanding of the ecdysone transduction network in the fruit fly.

Steroid hormones provoke large, long-term effects in animals. Some are homeostatic or physiological (*e.g.*, control of osmoregulation, immune system modulation), while others are developmental (*e.g*., the control of sexual maturation in vertebrates). Among the most striking of these developmental effects is the coordination of insect development and metamorphosis by the steroid molting hormone 20-hydroxyecdysone (20E, hereafter “ecdysone”). At metamorphosis, virtually every cell responds to the hormone, but the responses are as diverse as the adult organism that is being formed: many cells that played important roles during larval life are stimulated to start down pathways leading to programmed cell death, while others, destined to form adult tissues, enter pathways leading to differentiation and morphogenesis (for example, Li and White 2003). In humans, failures in steroid signaling cause diseases as diverse as mood disorders and oncogenesis. In *Drosophila melanogaster*, defects in ecdysone signaling lead to a complete failure of metamorphosis.

Hormones act by binding to nuclear hormone receptors (NHRs), which are deeply conserved across metazoans. These NHRs share a common structure: an unconserved N-terminal A/B region including a transcriptional activation domain (AF-1), followed by a highly conserved 66-68 residue DNA binding domain (DBD), a short hinge region, a conserved ligand binding domain (LBD), and unconserved sequences of variable extent called the F domain. Receptors function by binding to highly conserved response elements (HREs), where they act as powerful transcriptional effectors.

Ecdysone acts through a heterodimeric NHR composed of the products of the genes *EcR* and *ultraspiracle* (*usp*). EcR binds hormone only as part of the heterodimer (Yao *et al.* 1993), thus USP is an allosteric regulator with respect to ligand binding by EcR; similarly, DNA binding modifies ligand binding by the heterodimer (Azoitei and Spindler-Barth 2009). The “canonical” EcR/USP response element (EcRE) is an inverted repeat 5′-AGGTCA/TGACCT-3′ (Cherbas *et al.* 1991), but EcR/USP also binds direct repeats and inverted repeats of diverse spacing (D’Avino *et al.* 1995; Braun *et al.* 2009; Nakagawa and Henrich 2009). EcREs are known to be present throughout the genome (Cherbas *et al.* 1988; Koelle *et al.* 1991; Yao *et al.* 1993).

Numerous EcR/USP coregulators have been identified. Davis *et al.* (2011) carried out a bioinformatic search looking for potential coregulators based on the LXXLL motif common to many hormone receptors. Trithorax-related (TRR) is known to interact with EcR/USP and to methylate H3K4 (Sedkov *et al.* 2003). Cryptocephal (*Drosophila* ATF4) is known to interact directly with isoform B2 (Gauthier *et al.* 2012), Taiman (TAI), a p160 homolog, and Alien, a corepressor, colocalize with the receptor (Dressel *et al.* 1999; Nakagawa and Henrich 2009). There is evidence implicating the products of *Rig*, *Ash2*, β*Ftz-F1*, and the histone chaperone DEK as coregulators or critical components of coregulator complexes (Zhu *et al.* 2006; Sawatsubashi *et al.* 2010; Carbonell *et al.* 2013). *Drosophila* SMRTER (Smr, a relative of SMRT and NCoR) is known to be crucial to ligand-independent repression (Tsai *et al.* 1999; Sedkov *et al.* 2003). There is ample evidence that remodeling factors, including SWI/SNF and the NURF complex, interact with EcR/USP and play key roles in ecdysone response (Badenhorst *et al.* 2005; Zraly *et al.* 2006; Ables and Drummond-Barbosa 2010; Kugler *et al.* 2011; Zraly and Dingwall 2012). There is also evidence that ecdysone-induced expression is associated with acetylation of H3K23 (Bodai *et al.* 2012).

Typically, steroids induce (and/or repress) a limited number of direct coding and noncoding target genes including transcription factors (TFs) and microRNAs (Garbuzov and Tatar 2010). These responses ramify within hours, leading to secondary effects that may implicate thousands of genes and major changes in cell state. This pattern holds true for the ecdysone response; indeed, some of the earliest studies of primary and secondary responses during steroid stimulation described the changes in salivary gland puffing patterns at the onset of metamorphosis (Ashburner 1973; Yao *et al.* 1993). It has since become clear that at least one-fifth of *Drosophila* genes respond to ecdysone in some cell at one stage or another, according to previously published transcriptome-wide studies in limited tissues or cell lines (Beckstead *et al.* 2005; Gauhar *et al.* 2009; Gonsalves *et al.* 2011; Shlyueva *et al.* 2014). The number of responders in any one cell at any particular stage is much smaller. Because the effects of the hormone are global and the hormone is distributed systemically, the nature of an individual cell’s stage-specific response varies greatly (Andres and Cherbas 1992, 1994). Among the wide array of specific cellular effects are modulation of the cell cycle (Fallon and Gerenday 2010), induction of apoptosis (Cakouros *et al.* 2004; Kilpatrick *et al.* 2005), and neurite elongation (Tominaga *et al.* 2010).

These observations frame a central question: In any one cell, at any one stage, how are responding genes selected from the broad array of potential targets? Few genome-wide studies have been conducted of the ecdysone response. Following initial work using subsets of genes and microarrays (Beckstead *et al.* 2005; Gonsalves *et al.* 2011), Gauhar *et al.* (2009) employed low-resolution methods (enzymatic tagging) to provide initial data of the receptor binding sites in Kc167 cells and identified ecdysone-responsive genes. Kellner *et al.* (2012) showed that JIL-1 kinase is present at both enhancers and promoters of ecdysone-induced genes in (Kc167 cells) and argue that it phosphorylates nearby histone H3. They found that JIL-1’s presence is required for CREB-induced acetylation of H3K27 and is also required for recruitment of the 14-3-3 scaffold protein that is involved in multi-protein regulation. Shlyueva *et al.* (2014) performed the STARR-seq assay that identifies regions with enhancer activity in S2 and OSC cell lines before and 24 hr after ecdysone exposure. RNA-seq was performed in S2 cells before and after 24 hr of ecdysone exposure. These studies together provide a set of 3415 ecdysone-responsive genes from genome-wide ecdysone exposure studies from a small set of two cell lines (S2 and Kc) and two organ cultures (salivary gland and third instar larvae organs).

The catalog of early transcriptional responses to ecdysone in 41 *Drosophila* cell lines presented here sheds light on the regulatory rules active in hormone signal transduction. In previous work as part of modENCODE (Cherbas *et al.* 2011), we demonstrated that this collection of cell lines represents transcriptionally diverse states. Here, we find that genes that respond immediately to ecdysone differ substantially as a function of initial cell state. The vast majority of responsive genes respond in one or a few cell lines. A limited set of genes, including many previously known responders, is responsive in the majority of lines. Remarkably, numerous genes have the property that they are induced in some lines, repressed in others, and nonresponsive in still others – indicating that initial state can modulate the direction of response, as well as the cohort of responsive elements. We observe that the count of genes induced at 5 hr is highly correlated with the steady-state expression level of *EcR*, and not *usp* or any other TF, suggesting that *EcR* expression level is rate-limiting in the early response. We find that responsive genes cluster in neighborhoods, and that tandem arrays of genes on the same strand are more likely to be jointly induced than pairs of genes transcribed from bidirectional promoters. Lastly, we have identified a network of TFs that explains much of the variation in these responses. Given the virtues of clonal cell populations for both expression and mechanistic studies of regulation, this study establishes that the collection of *Drosophila* cell lines is a valuable setting in which to elucidate the dynamics of hormone signaling in a metazoan.

## Materials and Methods

### Cell lines and ecdysone treatment

The individual cell lines are listed in Table 1. All cell lines used in this study were obtained from the *Drosophila* Genomics Resource Center (DGRC, dgrc.cgb.indiana.edu), and grown as recommended there. Each cell line used in this paper has a formal name, which is the unique identifier used by FlyBase and the DGRC. For convenience, we use a shortened version of many of these cell line names in this paper; both the formal names and the short names are given in Table 1.

**Table 1:** Cell lines used in this study

Short Name of Cell Line	Formal Name	Tissue of Origin	Ref.	Features	Total Read Depth (M)
1182-4H	1182-4H	Embryo	1	FCB	136.15
CCa	CCa	Embryo	2	MD	210.38
L1	CME L1	Prothoracic leg disc (L3)	3	MVCBI	108.1
Cl.8	CME W1 Cl.8^+^	Wing disc (L3)	3	MVCI	131.06
W2	CME W2	Wing disc (L3)	3	MVCB	107.09
D1	D1	Embryo	4	M	83.76
DX	DX	Embryo	2	A	148.5
E-CS	E-CS	Embryo	5	F	146.78
E-OR	E-OR	Embryo	5	M	123.98
G1	G1	Embryo	6	M	95.83
G2	G2	Embryo	6	M	140.4
GM2	GM2	Embryo	7	MCB	142.59
GM3	GM3	Embryo	7	M	91.36
Jupiter	Jupiter	Embryo	8	M	63.53
Kc	Kc167	Embryo	9	FVCBDIE	197.17
mbn2	mbn2	Hemolymph (L3)	10	MCB	62.92
MCW12	MCW12	Wing disc (L3)	11	FVD	143.57
ML83-26	ML83-26	Embryo	12	F	123.78
BG1-c1	ML-DmBG1-c1	CNS (L3)	13	AVCB	114.13
BG2-c2	ML-DmBG2-c2	CNS (L3)	13	MVCBI	147.65
BG3-c2	ML-DmBG3-c2	CNS (L3)	13	MVCDIE	320.94
D1-c4	ML-DmD1-c4	Wing disc (L3)	14	MV	80.78
D11	ML-DmD11	Eye-antennal disc (L3)	14	MVCBI	188.04
D17-c3	ML-DmD17-c3	Haltere disc (L3)	14	FVCB	86.2
D20-c5	ML-DmD20-c5	Antennal disc (L3)	14	MVCBI	183.64
D21	ML-DmD21	Wing disc (L3)	14	MVCBI	105.42
D23-c4	ML-DmD23-c4	Wing disc (L3)	14	MV	98.39
D4-c1	ML-DmD4-c1	Mixed imaginal discs (L3)	14	MVCBI	147.7
D8	ML-DmD8	Wing disc (L3)	14	FVCB	223.87
D9	ML-DmD9	Wing disc (L3)	14	AVCB	194.2
OSS	OSS	Ovary (Adult)	15	FVPB	113.75
PR-8	PR-8	Embryo	16	MR	173.2
Pten X	Pten X	Embryo	16	M	210.2
Ras-wts:RNAi	Ras[v12];wts[RNAi]	Embryo	17	MR	105.12
Ras-H3	Ras[v12]-H3	Embryo	18	MR	163.44
Ras-H7	Ras[v12]-H7	Embryo	18	MR	189.44
Rumi-Ras	Rumi[26]Ras[v12]-4	Embryo	19	FR	119.24
S1	S1	Embryo	20	MCB	126.57
S2-DRSC	S2-DRSC	Embryo	20	MCE	102.67
S3	S3	Embryo	20	MCB	123.66
Sg4	Sg4	Embryo	20	MCB	85.86

References: 1, Debec 1978; 2, V. Gvozdev, personal communication; 3, Currie *et al.* 1988; 4, A. Dubendorfer, personal communication; 5, Bernhard *et al.* 1980; 6, W. Gehring, personal communication; 7, Mosna and Dolfini 1972; 8, Karpova *et al.* 2006; 9, Echalier and Ohanessian 1969; 10, Gateff *et al.* 1980; 11, M. Milner, personal communication; 12, T. Miyake, personal communication; 13, Ui *et al.* 1994; 14, Ui *et al.*, 1987; 15, Niki *et al.* 2006; 16, Justiniano *et al.* 2012; 17, Simcox *et al.* 2008; 18, Dequeant *et al.* 2015; 19, Leonardi *et al.* 2011; and 20, (Schneider 1972). Features: F, female; C, transcriptome described in Cherbas *et al.* 2011; B, transcriptome described in Brown *et al.* 2014; M, male (M, F, or A assignment from Lee *et al.* 2014 or by the same criteria using data from this paper; A, ambiguous gender; *P*, the parental line fGS/OSS is a coculture of somatic sheath and germ cells [OSS was generated from fGS/OSS by the loss of the germ cell component (Niki *et al.* 2006)]; D, duplicated in RNA-seq analysis; V, variation to standard medium (M3+BPYE with heat-inactivated fetal calf serum, particular variations are noted in Table S1); I, analyzed by microarray in preliminary ecdysone study; E, analyzed in extended ecdysone response time course (0, 1, 3, 5, and 7 hr); R, cell line expressing constitutively active Ras85.

All of the cell lines were of independent origin. S2 cells have been grown for many years in a large number of laboratories, under variable conditions, and different isolates from the same original line (Schneider 1972) often have widely different properties (Y. Zou and L. Cherbas, unpublished results); two isolates of largely unknown provenance (S2-DRSC and Sg4) were used in the experiments reported here. Kc was also grown in multiple laboratories for a number of years after it was cloned. All of the other lines were obtained by the DGRC from the laboratories in which they were established and can be assumed to have been maintained carefully with a limited number of transfers. All experiments were performed on cells in exponential growth.

Cells were treated with 20-hydroxyecdysone (10^−6^ M final concentration, Sigma-Aldrich) as described previously (Savakis *et al.* 1980). RNA was extracted with Trizol (Invitrogen) according to the manufacturer’s protocol; in a few cases the RNA was further purified on RNeasy columns (Qiagen). A full description of the procedure can be found in (Eads *et al.* 2006). Samples were sequenced on Illumina Hi-seq machines and produced single-end, unstranded, 100 bp reads.

### Differential expression analysis

Gene and exon level counts were computed using the python package HTSeq (Anders *et al.* 2015) (version 0.6.1p1) using the FlyBase annotation version r5.57 (St Pierre *et al.* 2014). Exon level counts were analyzed using the DEXSeq R package (Anders *et al.* 2012, version 1.12.1). Exon level ecdysone exposure effects are reported only for a model fit across all cell lines. Thus, exon p-values and fold changes are not reported for each cell line individually. Gene level analysis was completed using the DESeq R package (Anders and Huber 2010, version 1.18.0). As only a portion of the samples were completed in biological duplicate, gene level dispersion estimates were made using the replicated samples and applied to all cell lines. The statistical assumption underlying this analysis is that gene-level biological dispersion is consistent across cell lines. Reported log2 fold change and p-values are reported in Supporting Information, Table S5.

### Identification of widespread and restricted ecdysone-responsive genes

In order to leverage the breadth of cell lines examined in this study while identifying ecdysone-responsive genes, we employed a threshold based on the responsiveness across all cell lines. This threshold allows genes that show a trend toward significance, while possibly not achieving standard statistical significance within any particular cell line, to be confidently identified as ecdysone-responsive. Formally, this is measured by the Fisher’s Method test for trend in significance across all cell lines.

Each gene is associated with two Fisher’s Method values corresponding to a trend toward induction and repression across all cell lines. We note that it is possible to achieve significance in both responsive behaviors under this structure. The procedure to produce these tests is analogous for induction and repression, so we will describe the procedure for induction here. In order to compute a Fisher’s Method significance value, the p-values produced by the differential expression analysis are used to construct a ranked list of induced genes within each cell line. Genes that are repressed are assigned a p-value of one and thus are tied at the bottom of that cell line’s rank list. These rank values thus represent a uniform marginal distribution for each cell line, as required in order to apply Fisher’s Method. For each gene, the rank within each cell line is combined using Fisher’s Method. Genes that tend toward the top of the rank list in many cell lines will produce significant values, while genes randomly distributed among each list will produce less significant values.

Two types of thresholds, biological relevance and statistical significance, are applied to each gene within each cell line. The biological relevance threshold is defined by a fold change upon ecdysone exposure greater than twofold (inductive or repressive). Statistically significant responsive genes are those that achieved either an adjusted p-value less than 0.01 regardless of Fisher’s Method p-value, or an unadjusted p-value of 0.01 and a Fisher’s Method p-value less than 10^−8^. These genes identified as significantly ecdysone-responsive in a particular cell line can be found in Table S6.

### Enrichment of motifs

Enrichment of motifs within promoter region DNA sequences was carried out using the homer2 program (Heinz *et al.* 2010) with the “known” command against the supplied all.motifs database, which contains the EcRE motif of interest. Scripts and database are available online http://homer.salk.edu/homer/motif/.

### Summary analysis

All statistical analyses were computed using R (version 3.1.2) using custom scripts. Gene lengths for length-normalized expression were taken as the mean of the lengths of the transcripts for that gene. GO term enrichment was produced using the fb_2014_03 version of the FlyBase gene ontology (Gene Ontology *et al.* 2013). Only genes with at least one annotated ontology term were used for enrichment calculates. All GO term enrichment p-values were calculated using the hypergeometric distribution.

### Responsive proximal genes

For all significantly responsive genes, the fraction of responsive genes and the average response direction of nearby genes were analyzed. In order to determine the distance between two genes, the ecdysone relevant transcription start sites (TSS) were first determined. For genes with multiple transcription start sites, the TSS was determined to be the TSS associated with a significant exon level response to ecdysone if one exists, or the exon with the highest length normalized expression in the relevant time point (*i.e.*, 5 hr time point for induced genes, 0 hr time point for repressed genes, or the average for nonresponsive genes). The selected transcription start sites are included in Table S5.

Each gene within 20,000 base pairs of a responsive gene was associated with two values, first if the gene was responsive and second the direction of response, taken as the negative log10 of the p-value multiplied by the sign of the log fold change after ecdysone exposure divided by before (these values are trimmed to plus and minus 10 to avoid outlier effects). A moving window of 100 gene–cell line combinations was used to calculate the fraction of responsive genes and the average response direction within each bin. The binned points were grouped according to the response direction of the responsive gene as well as the shared promoter architecture (upstream/downstream and same/opposite strand). These points were then smoothed using LOESS with a local linear fit over binned points. The plot produced is found in Figure 3.

### Restricted ecdysone-response prediction

In order to predict the restricted response, the following two models (referred to as matched induction and matched repression) were fit. The models are symmetric, so only the matched induced model will be described in full detail.

At each gene in the restricted induced set, we aimed to predict which cell lines responded significantly and which responded most repressively, defined as the cell lines showing the smallest log2 fold change of ecdysone exposure expression/preexposure expression. A matching number of the most repressive responsive cell lines were chosen such that each gene contained a balanced number of induced and matched repressed cell lines.

In order to predict which gene–cell line combinations belonged to the above described sets, the model was provided with normalized TF expression masked by a known TF motif from the TOMTOM database (Gupta *et al.* 2007), present in the promoter of the gene to be predicted. TF expression was included only if at least one cell line expressed the TF above the 20th percentile of genes with at least one read. A TF motif was considered significant by setting the threshold on the allowed mismatches to the known PWM, such that just less than 5% of nonresponsive genes’ promoters contained a hit to the motif. There were 270 TFs with known motifs and valid expression. Additionally, the model was given the rank of the normalized expression of the gene to be predicted, as lowly expressed genes are intuitively less likely to be repressed and more likely to be induced, and conversely for highly expressed genes.

This model was then fit using the random forests model (Breiman 2001; Abraham *et al.* 2014). Important variables were determined from a model fit on all 41 cell lines. Accuracy measures were obtained by constructing the above outcome and predictor variables and then removing each cell line as a test set. The data from the remaining cell lines were used to train the random forest and the data from the left out cell line was used to test the accuracy of the model’s predictions. Accuracy measures from all cell lines were averaged and reported.

In order to determine the effect of increased numbers of cell lines on prediction accuracy, samples of cell lines were taken randomly and restricted responding genes, outcome, and predictor data were constructed. The left out cell line method was again used to determine the accuracy of the model. Since there were many subsets of cell lines that could be chosen, the subsetting procedure was repeated 1000 times for each number of cell lines and all average accuracy values are reported.

### Extended time course analysis

For a subset of three cell lines (Kc, BG3, and S2) that have an extended time course, including the 0, 1, 3, 5, and 7 hr time points, the following analysis pipeline was conducted. In order to compare the transcriptional responses across the time course for each cell line, a normalization was performed that allows comparison of genes with dissimilar steady state expression levels, but may share ecdysone response “shape.” This normalization begins by applying the robust median library size normalization from the DESeq R package (Anders and Huber 2010). Then, a mean centering is applied at each gene and cell line across all five time points. The gene–cell line expression is then divided by the fitted standard deviation across all gene–cell line combinations, in order to adjust for the known increase in variance at higher expression loci. These normalized expression measures can thus be interpreted as a response shape across time for each cell line and the response shape is comparable for genes at different mean expression levels, but the trend and relative scale of response over time is maintained. Units represent standard deviations from the mean across time. These normalized expression values are analyzed in the context of the response at the 5 hr time point.

### Data availability

All sequences are available through the SRA (BioProject PRJNA306537) and individual sample accession numbers are available in Table S2 Micraarray data re in the GEO database at NCBI as series GSE11167.

## Results

### Overview of study design

RNA samples were collected from 41 cell lines (Table 1 and Table S1) before and after a 5 hr exposure to ecdysone at a biologically relevant concentration of 10^−6^ M. Transcription levels were measured by single-end poly(A)^+^ RNA-sequencing with 100 bp reads. For four of the cell lines, CCa, Kc, MCW12 and BG3-c2, duplicate samples were collected in order to estimate the biological variation present in this system. Samples were taken from an extended time course of exposure at 1, 3, 5, and 7 hr for three cell lines, Kc, BG3-c2 and S2-DRSC. All sequences are available through the SRA (BioProject PRJNA306537) and individual sample accession numbers are available in Table S2. Gene and exon-level transcriptional quantifications were assessed using the FlyBase r5.57 annotation (Table S3 and Table S4). Differential expression calculations were carried out using the DESeq (Anders and Huber 2010) R package (see *Materials and Methods*). Significantly induced or repressed genes were identified by applying a biological relevance fold change threshold of two and a statistical significance threshold of 0.01 adjusted p-value. A relaxed statistical significance threshold of 0.01 unadjusted p-value was applied for genes that showed a strongly significant tendency for induction or repression across many cell lines (Fisher’s method p-value < 10^−8^, see *Materials and Methods*). Differentially expressed (DE) genes along with meta-information can be found in Table S5 and Table S6. (Note that Table S5 is interactive and enables the exploration of the effects of alternative thresholds.)

Additionally, microarray experiments examined 12 cell lines (control and 5 hr after ecdysone treatment) using DGRC-2 microarrays (oligonucleotides representing 64,020 exons from 14,518 genes as annotated in FlyBase v 2010-02). Effects were scored as significant at an FDR threshold of 1%. All results have been deposited in GEO. Data were biologically replicated in triplicate and results can be found in Table S5 along with matched RNA-seq results.

### Diverse cell states profiled

Cell lines are remarkably transcriptionally diverse (Cherbas *et al.* 2011). More than 70% of all *Drosophila* genes (11,884) are expressed at greater than one RPKM (reads per kb per million mapped reads) in at least one of the 41 cell lines we profiled at ground state (prior to ecdysone exposure). Of those, 5846 are constitutively expressed in all cell lines, while 1459 are expressed in only a single cell line. The number of expressed TFs per cell line ranges from 406 to 450, and 595 (85% of all TFs in fly, Hammonds *et al.* 2013) are expressed in at least one cell line. Further, TFs are expressed at quantitatively different levels in each cell line (Figure S1). After ecdysone exposure, an additional 305 genes are expressed that were not basally detectable in any cell line and, conversely, 360 genes are constitutively inactivated after ecdysone exposure. We defined genes with significant responses in more than half of the cell lines as “widespread,” and others as “restricted.”

**Figure 1:**
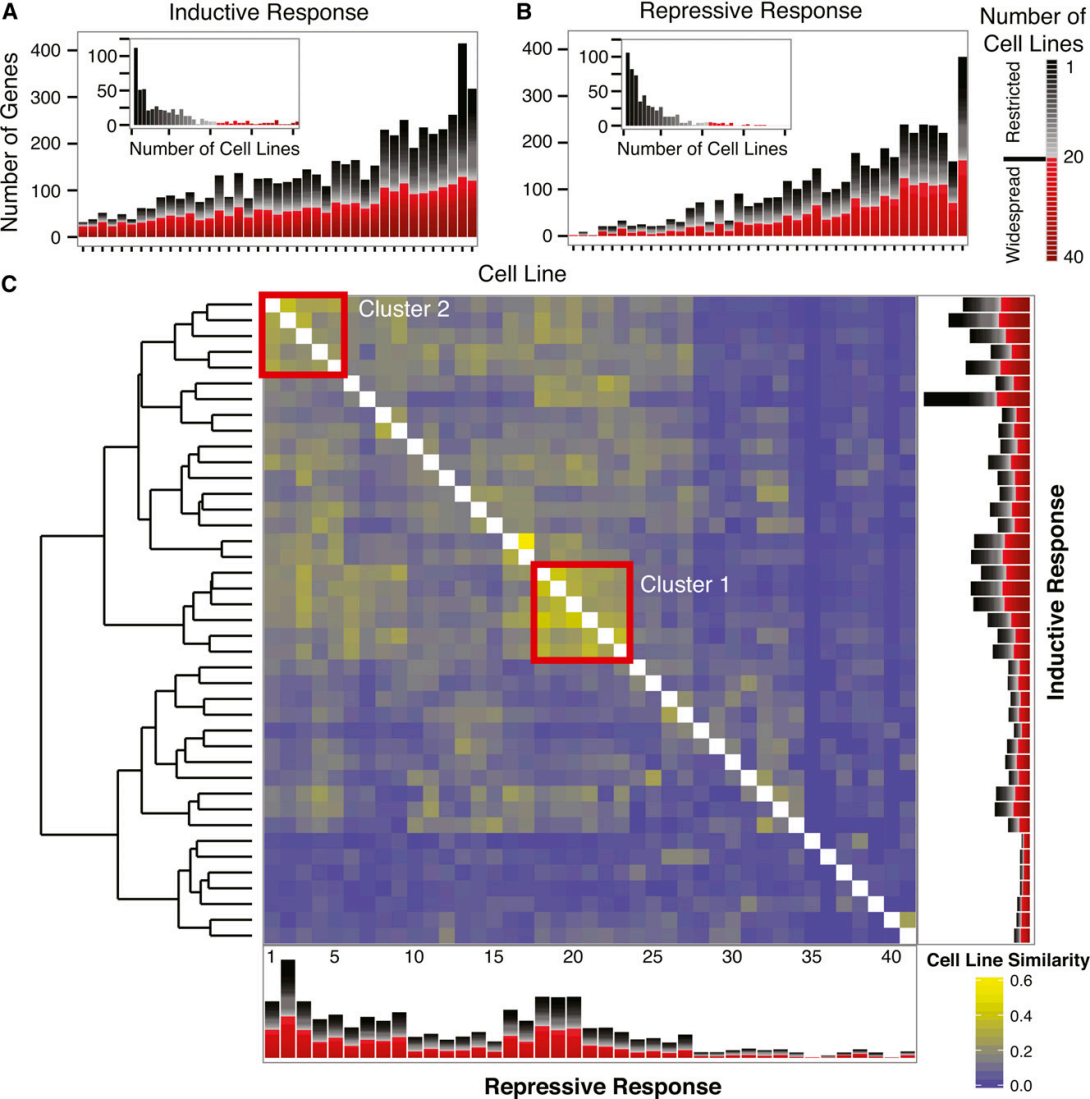
Cell line responses: breadth and similarity. Using the thresholds defined in the text, the inductive (A) and repressive (B) response within each cell line is represented. The red shaded bars represent widespread genes (responsive in more than half of cell lines) and black shaded bars indicate restricted response genes (responsive in less than half of cell lines), as noted in the legend. Main histograms show the response of each cell line, ordered by total number of responsive genes E-OR, Ras-H3, D11, BG3-c2, D23-c4, D21, D8, MCW12, Sg4, S3, ML83-26, PR-8, D4-c1, D20-c5, G1, Rumi-Ras, Pten X, Jupiter, Ras-H7, GM3, D1-c4, 1182-4H, S2-DRSC, S1, CCa, W2, L1, D9, E-CS, DX, Cl.8, Kc, mbn2, BG1-c1, GM2, OSS, D1, D17-c3, BG2-c2, G2, and Ras-wts:RNAi. Inset histograms show the number of cell lines in which each gene is responsive. (C) Cell line similarity (Jaccard similarity) within the restricted response is used to cluster the cell lines as shown in the dendrogram on the left. Repressive (lower left) and inductive (upper right) response similarity corresponds to the scale indicated on the lower right. Stacked barplots in C are the same as those in A and B, reordered by the hierarchical clustering dendrogram into the following order (from left to right): D11(1), E-OR, D21, ML83-26, MCW12(5), Pten X, Ras-H3, Rumi-Ras, PR-8, 1182-4H(10), D20-c5, D1-c4, GM3, G1, S2-DRSC(15), Sg4, S3, D8, BG3-c2, D23-c4(20), D4-c1, Ras-H7, Jupiter, D9, W2(25), E-CS, S1, GM2, Kc, DX(30), Cl.8, L1, CCa, mbn2, Ras-wts:RNAi(35), G2, D17-c3, OSS, D1, BG2-c2(40), and BG1-c1.

The vast majority of restricted genes are expressed in only a few cell lines; only 100 comprise the widespread class (Figure 1, A and B). Indeed, few pairs of cell lines overlap in their restricted responses by more than 20% (Figure 1C and Figure S2). The two most striking clusters of cell lines (cluster 1: D8, BG3-c2, D23-c4, D4-c1, Ras-H7, and Jupiter, and cluster 2: D11, E-OR, D21, ML83-26, and MCW12; emphasized in Figure 1 by red boxes) do not share physiological origin, sex of the cells, or other covariates as seen in Figure S3. In general, the response to ecdysone across these cell lines does not appear to be well correlated with common cell line characteristics. Therefore, this *in vitro* system provides the opportunity to study diverse and distinct ecdysone response dynamics as a function of initial transcriptomic cell states.

We note that the collection of cell lines includes six created by expression of a constitutively active Ras oncogene (Ras-H3, Ras-H7, Ras-wts:RNAi, and Rumi-Ras), a null mutation in the tumor suppressor gene Pten (PtenX), or both (PR-8) (Simcox *et al.* 2008; Justiniano *et al.* 2012). Four of these lines cluster when ecdysone responses are compared (Figure 1 and Figure S3). An additional activated Ras line (Ras-H7) shows some similarity in its responses. If confirmed, the existence of a Ras/Pten cluster could suggest that the pathway to generation of a cell line by either gain of Ras function or loss of Pten function leads to a transcriptional environment conducive to a particular pattern of ecdysone response.

### Ecdysone receptor is rate-limiting for global responsiveness

A total of 1645 genes are significantly transcriptionally responsive in at least one cell line. Fifty-nine TFs are induced in response to ecdysone, and 35 of these are responsive in five or fewer cell lines (Table S5). Several of these are known ecdysone-responsive TFs, while many are newly identified and point to new hormone-responsive pathways. Table S7 provides enriched GO terms among induced and repressed genes.

It is well known that some tissues and cell types are more responsive to ecdysone than others. We measured the responsiveness of a cell line as the count of genes significantly induced or repressed 5 hr after induction, and refer to this as the Responsive Gene Count (RGC). While RGC is threshold-dependent, the rank-order of cellular responsiveness is well preserved across a broad range of biologically and statistically meaningful parameterizations (see *Materials and Methods*, Table S8). The RGC varies by two orders of magnitude across cell lines and is driven by responsive genes with highly restricted expression patterns (Figure 1 and Figure S4).

**Figure 2:**
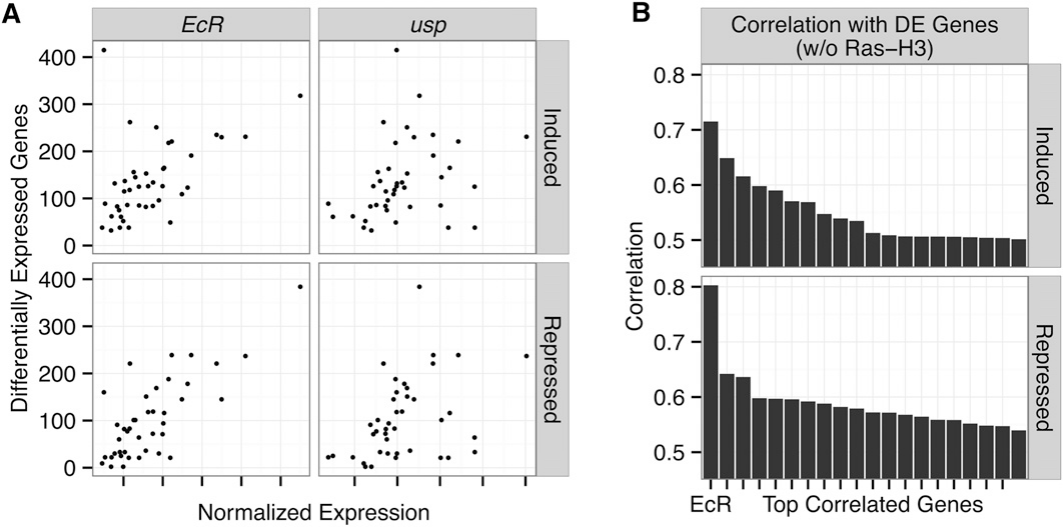
Ecdysone receptor expression correlation. (A) Scatter plots comparing the responsive gene count (RGC) of induced (upper panels) and repressed (lower panel) genes with the normalized expression of the canonical ecdysone heterodimer receptor (*EcR* and *usp* panels). (B) Barplots showing the correlation of normalized expression with number of induced or repressed genes for the genes with the 20 highest correlations (genome-wide). *EcR* shows the highest correlation for both induced and repressed genes. A full ranked list of correlations with number of responsive genes can be found in Table S9.

The RGC does not correlate with the count of basally expressed TFs (r ∼ –0.04, p = 0.85) and correlates only weakly (and inversely) with the total number of genes expressed per cell line (r ∼ –0.35, p = 0.03). We assessed the association of the RGC with the basal expression level of each gene in the genome in a multiple testing setting (see *Materials and Methods*). Among all genes, the expression level of *EcR* is by far the most strongly correlated with RGC (r ∼ 0.71, FDR < 0.001, minimum FDR for other genes > 0.03, Table S9). We further assessed this observation within both induced and repressed genes (Figure 2) and found that *EcR* expression level is strongly correlated with both. In fact, *EcR* is the only gene statistically significantly correlated with the number of both induced and repressed genes (*EcR* FDR < 0.001, minimum FDR for other genes > 0.02). The *EcR* heterodimer partner, *usp*, exhibits weak correlation with RGC (r > 0.35, FDR > 0.9; Figure 2).

The residuals left after correcting for the effect of *EcR* expression level on RGC, as well as on the counts of induced and repressed genes (separately), show little correlation with any gene (maximal correlation < 0.61; minimum FDR > 0.6). Taken together, these results indicate that *EcR* titer is rate-limiting for the transcriptional response to ecdysone.

### Common ecdysone response: the widespread set

The 100 genes with widespread responses include 68 induced and 32 repressed in more than half of our cell lines. Five genes, *Hr4*, *Hormone receptor-like in 46* (*Hr46*), *Ecdysone-induced protein 75B* (*Eip75B*), *CG44004*, and *bip1*, are induced in all 41 cell lines. All five have been previously identified in other genome-wide surveys of ecdysone exposure response (Beckstead *et al.* 2005; Gonsalves *et al.* 2011; Gauhar *et al.* 2009; Shlyueva *et al.* 2014) (Table S10). There are no genes repressed in all cell lines, with *fruitless (fru)* being the most widespread (repressed in 33 of 41 cell lines). More broadly, widespread induced genes are significantly enriched for biological GO terms “metamorphosis,” “salivary gland cell autophagic cell death” and “steroid hormone mediated signaling” (p-value < 0.001), as expected, since the majority of these genes have been previously reported in other studies of ecdysone response (Table S10).

The promoter regions of widespread induced genes, defined as 500 bp upstream of each TSS, are 36% enriched over background for the EcRE motif (p-value < 0.02), representing the most significantly enriched of all known motifs in the HOMER library (Heinz *et al.* 2010) (see *Materials and Methods*).

Notably, 11 widespread induced genes lack GO annotations, and these include four annotated long noncoding RNA (lncRNA) genes, *CR43432*, *CR43626*, *CR45391*, and *CR45424*. These lncRNAs are each expressed in the salivary gland and fat body, and at low levels in most other tissues. Only *CR43432* is expressed at high levels during development, with maximal expression (> 100 RPKM) in the 4–14 hr embryos, consistent with response to the midembryological ecdysone pulse. This is in contrast to the majority of lncRNAs in *Drosophila* (and indeed mammals), which are expressed predominantly in tissues of the nervous system and the gonads (Brown *et al.* 2014; Derrien *et al.* 2012). Notably, this lncRNA is induced at levels comparable the well-known response *polished rice*, which encodes short (11 amino acid) peptides critical for ecdysone transduction in the epidermis (Chanut-Delalande *et al.* 2014). *CR43432* encodes three short, ultraconserved ORFs, and hence constitutes a candidate protein-coding gene.

The set of widespread repressed genes is much smaller than the set of induced genes, as is the overall repressive response in most cell lines. There are no statistically enriched GO terms among this set of genes.

### Diversity of the ecdysone response: the restricted set

We find that 93% (863) of induced genes and 96% (756) of repressed genes are affected by ecdysone in fewer than half our cell lines. These restricted responses form the molecular basis of the diverse transcriptional and physiological effects that the hormone induces throughout development and within distinct cell types. A total of 400 and 241 genes are induced and repressed, respectively, in exactly one cell line, with a large fraction (31%) responding only in the Ras-H3 cell line, an outlier in this study (see *Materials and Methods*).

Eighty-three genes are significantly induced in some cell lines and significantly repressed in others (Table S11). Sixteen are induced and repressed in at least two cell lines. One striking example is the TF *CG9932*, which is significantly induced in five cell lines and significantly repressed in six. *CG9932* is differentially expressed across development with peaks at 20 hr and late L3 stage and shows strong expression in embryonic fat body and salivary glands. It is likely that some promoters respond in distinct directions based on prior epigenetic state. This phenomenon has also been noted in mammalian response to glucocorticoids (Chodankar *et al.* 2014).

The restricted set includes genes that respond weakly in several cell lines. Some of these genes do not pass our statistical criteria in any single sample, but by aggregating information across cell lines we obtain sufficient power to confidently annotate weak, reproducible induction or repression (see *Materials and Methods*). A total of 635 genes, 335 induced and 300 repressed, of this type are present genome-wide. Hence, leveraging our large number of cell lines we are able to confidently detect weak modulation of a large cohort of genes. These relatively small perturbations may be of no physiological significance to the cell; however, this may not always be the case. Weakly induced genes are strongly enriched for GO terms including “protein binding,” “vesicle-mediated transport,” and “macroautophagy,” and weakly repressed genes are enriched for “rRNA processing” and “calmodulin binding” (Table S7). Indeed, all five core TFIIH complex genes (and all nine components except *hay)* are weakly repressed in at least two-thirds of cell lines, while not a single gene is significantly responsive. While each of these individual modulations is small, the cumulative effect in a given cell on the titer of the functional TFIIH complex may be substantial. Genome-wide, weakly induced genes across most cell lines are found within 2.5 kb of a significantly induced gene more often than expected by chance (84% enrichment, binomial p-value <10^−5^). This pattern does not hold for weakly repressed genes.

### Responsive–proximal genes tend to respond similarly

**Figure 3:**
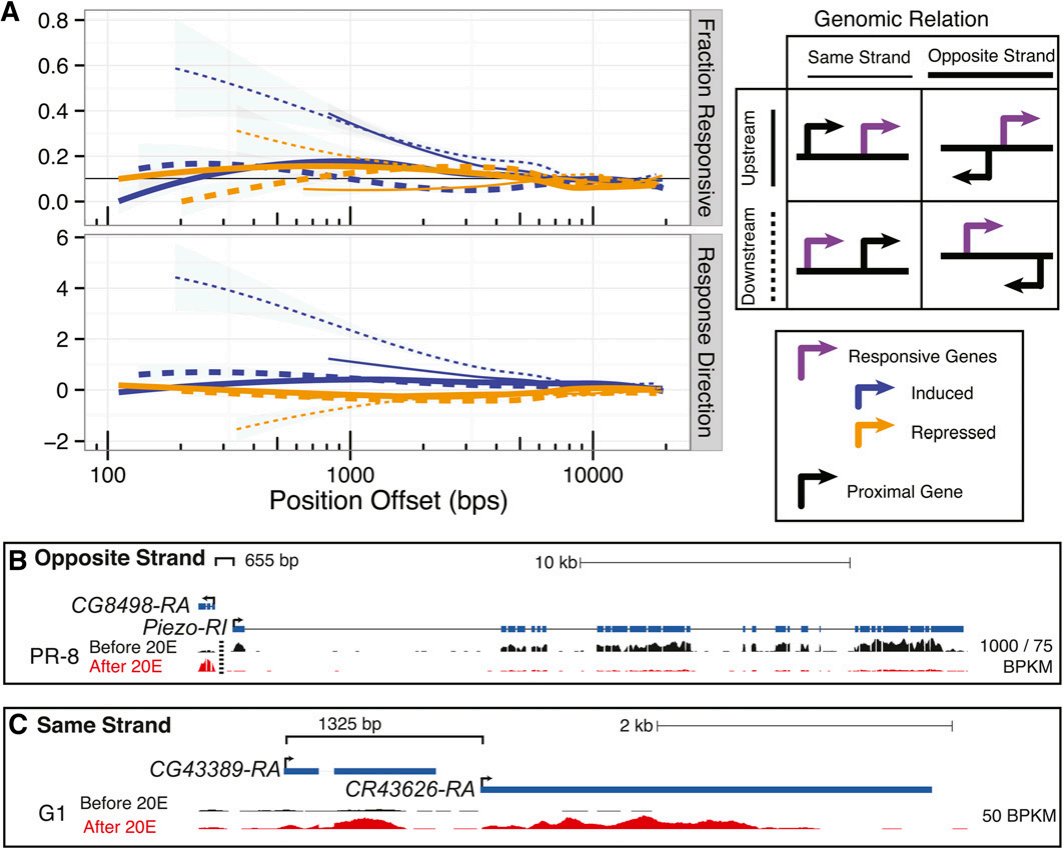
Genomic positional dependence of ecdysone response. (A) Each line represents the smoothed fraction of either responsive genes (upper panel) or response direction (lower panel). Response direction is measured by the mean of the magnitude and direction of response for genes within a moving window across genomic position. Genes within 20 kbp of a significantly responsive gene contribute to smoothed lines grouped by their genomic positional relation, see key. Genes proximal to a repressed or induced gene are summarized by red and black lines, respectively. In the first panel the blue line represents the genome-wide average as measured by the 10–20 kb proximal region. (B) The *CG8498* and *Piezo* locus is an example of divergent promoters responding in opposing directions. This exemplifies the trend shown in A where divergent promoters do not tend to consistently respond as strongly. Note that since *CG8498* and *Piezo* are expressed at different levels, the maximal height to the left of the vertical dashed line is 1000 RPKM and the maximal height to the left is 75 RPKM. (C) The *CG43389* and *CR43626* locus is an example of “operon-type” promoter structured genes responding in the same direction. This exemplifies the trend shown in A where “operon-type” promoters tend to respond consistently, particularly for induced genes.

Genes proximal to responsive genes tend to be responsive (p-value < 10^−15^; sample *t*-test against median for genes between 10–20 kb) and additionally tend to respond in the same direction (p-value < 10^−100^ induced and p-value < 10^−19^ repressed; one-sample *t*-test). The response of opposite-strand gene pairs (bidirectional and convergent) is less coordinated than genes with operon-type architecture (same strand pairs, Figure 3A and Figure S5, p-value < 10^−10^). One striking example of a divergently responsive bidirectional pair is *Piezo* and *CG8498*. The TSSs of these genes are separated by only 655 bp (Figure 3B) and the responses in most cell lines are strong and in opposite directions. *CG8498* is a widespread induced gene with 38 cell lines responding significantly, and all but two cell lines showing repression of *Piezo*. One example of coresponsive genes in an operon-type configuration is the pair *CG43389* and the noncoding gene *CR43626*, which are significantly induced in 32 and 35 cell lines, respectively (Figure 3C).

Taken together, these results indicate that response to ecdysone may involve or depend upon local chromatin organization or modification. Indeed, the gene encoding the bromodomain protein toutatis (tou), a protein involved in chromatin remodeling (Vanolst *et al.* 2005), is strongly induced in 15 cell lines. Both an acetyllysine binding domain (bromodomain) and a methyl-CpG binding domain exist in TOU. As a class, these BAZ (bromodomain adjacent to zinc finger) genes appear to be involved in the integration of information encoded in DNA methylation and posttranslational histone modifications (Filippakopoulos and Knapp 2014). The involvement of acytellysine binding factors is consistent with previous reports demonstrating that ecdysone transduction impacts H3K23 acetylation (Bodai *et al.* 2012).

### Isoform-specific ecdysone responses

Along with the gene level response to ecdysone we observe induction and repression of specific exons/isoforms in response to ecdysone, predominantly promoter-switching events, as has been previously reported (Shlyueva *et al.* 2014). The widespread responsive gene *Eip75B* shows the strongest exon level event in the genome across all cell lines (p-value < 10^−100^), consistent with previous reports (Bernardo *et al.* 2009; Shlyueva *et al.* 2014). Sequencing tracks and exon level expression analysis are found in Figure S6 and Figure S7. In total, 35 genes show significant exon level induction events and 31 genes show significant repression events (thresholds: adj. p-value < 0.01 and 50-fold change). Six genes, including previously reported events at *Eip75B* and *Eip74EF* and novel events at *Ect*, show significant induced and repressed exons representing promoter-switching events. Exon level statistics for significant events are given in Table S12.

### EcR isoforms

**Figure 4:**
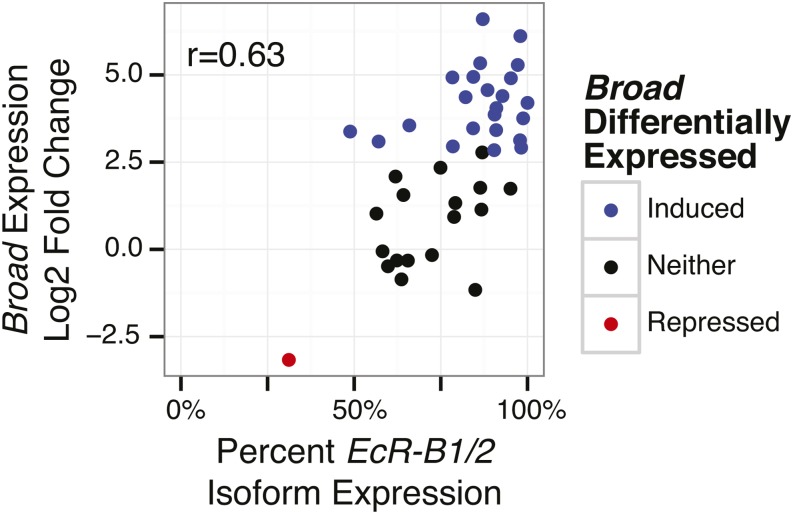
Ecdysone receptor isoform correlation. Scatter plot showing the correlation between EcR isoform expression ratio and the log2 fold change of *broad* (*br*) expression upon ecdysone exposure. The response of br shows the largest dynamic range among genes with the highest correlation to EcR isoform ratio.

Cherbas *et al.* (2003) demonstrated that the alternative *EcR* isoforms (*EcR-A*, consisting of transcripts A, D, and E; and *EcR-B1/2*, consisting of transcripts B, C, and G) play important roles in development. Gonsalves *et al.* (2011) showed that *EcR* isoforms are differentially expressed between Kc cells and salivary gland cells, indicating that alternate *EcR* isoforms elicit different transcriptional responses. Cell lines in this study show a wide range of *EcR* isoform expression. The length normalized fraction of the *EcR-B1/2* isoform expression (see *Materials and Methods*) ranges from 0.31 (BG3-c2 cell line) to 1 (S2 cell line) with most cell lines expressing predominantly the *EcR-B1/2* isoform. We do not see strong correlation to the total number of induced or repressed genes, or the residuals after correcting for the main *EcR* expression effect, but we do observe strong correlation between the *EcR-B1/2* isoform fraction and the expression of many individual genes. The most significantly correlated genes are *gliolectin* (*glec*), *squeeze* (*sqz*), *CG5059*, *Eip55E*, and *broad* (*br*) (top 100 genes are listed in Table S13). Only *glec* and *Xbp1* show increased expression with increased *EcR-A* isoform levels among the top 10 most correlated, consistent with a predominantly repressive role for *EcR-A* (Wilcox rank-sum p-value < 10^−10^). Of the highly correlated genes, the TF *br* shows the largest dynamic range (two orders of magnitude). Twenty-three cell lines show significant induction and one, BG3-c2, shows significant repression (Figure 4).

### Restricted responses depend in detail on the expressed cohort of TFs

While the global level of responsiveness (ERG) is well characterized by EcR titer, other effects are clearly at work producing diverse restricted responses. TFs are likely candidate EcR cooperative factors. We developed a statistical machine-learning model aimed at identifying these factors: we used basal expression levels and TF binding motifs to predict restricted responses. We also supplied the model with information about the basal expression level of each gene, since it is more difficult to detect repression of genes that are poorly expressed, and to detect induction of genes that are highly expressed.

We fit models for induction and repression. A notable feature of our modeling strategy is that we hold out entire cell lines, so when we assess our model’s predictive performance, it is assessed on cell lines it has not previously seen. This ensures that our model is generalizable to unseen cell lines. We find a predictive accuracy on held-out cell lines of 61% and 64% for induced and repressed genes, respectively, indicating that we have weak but significant power to predict the direction of a gene’s response.

We used feature selection to compute the relative importance of each covariate in our model (see *Materials and Methods*). We find that, for models of both induction and repression, the rank of basal gene expression level is the most important covariate. For the repression model, we see that gene rank is two and half times more important than the most important TF. In the induction model, the gene rank is only one and one-third times more important. However, in both models, several TFs also show comparably significant importance values (listed in Table S14). The most important TFs include known ecdysone response factors *br* and *Eip74EF*. TFs not previously implicated in the ecdysone response are also important; these include *longitudinals lacking* (*lola*) and *Chorion factor 2* (*Cf2*). We note nearly all covariates have weak positive values, indicating that the restricted response is a function of a large number of TFs, and hence that ecdysone response depends in detail on the expressed cohort of TFs.

This approach also enables us to assess the power of transcriptional profiling for elucidating the basis of hormone responses. We fitted and assessed this model successively using different numbers of cell lines, between 4 and 40 (Figure S8). From this analysis, we extrapolated a theoretical maximal accuracy (asymptote) for identifying the response of an unseen cell line of 74% and 79%, respectively, given an infinite number of cell lines in the training set (see *Materials and Methods*). Therefore, transcriptional profiling alone is not sufficient to fully elucidate the ecdysone response.

### Dynamics of extended temporal response

**Figure 5:**
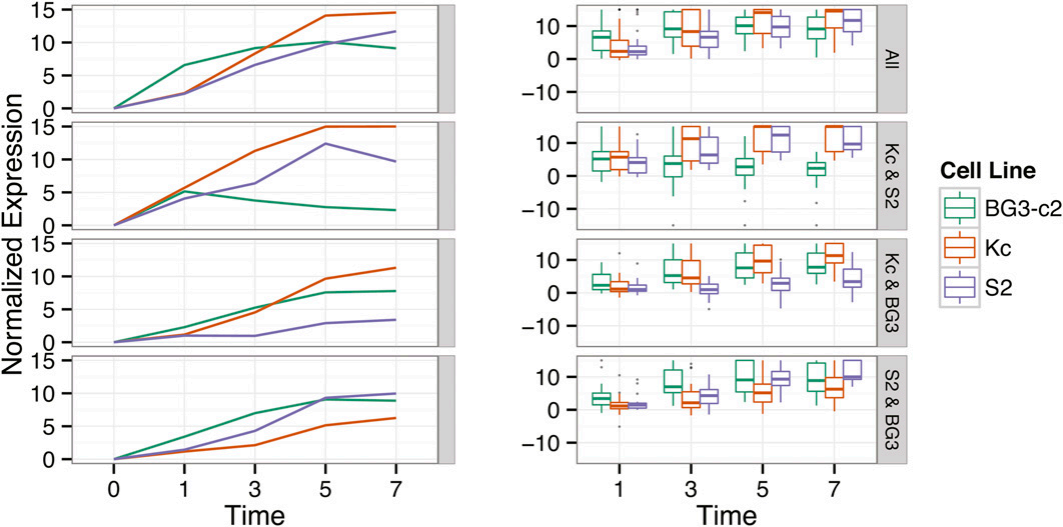
Extended time course. Four panels show normalized expression (see *Materials and Methods*) over an extended time course of 0, 1, 3, 5, and 7 hr for subsets of genes identified as significantly ecdysone-responsive in the 0–5 hr analysis for these three cell lines. The left panels show the median normalized expression at each time point and the right panels show the full set of normalized expression values at each time point. Sets of genes uniquely identified within each cell line as well as significantly repressed genes can be found in Table S4.

In addition to the response detected at the 5 hr time point, we explored the response to ecdysone for an extended temporal range, including 1, 3, 5, and 7 hr after exposure for three cell lines, BG3-c2, Kc and S2. We normalized responses, setting the basal expression level of each gene to zero, and then quantified changes at subsequent time points in multiples of fitted standard deviations (see *Materials and Methods*). This intuitive representation captures much of the same information as z-scores, and has the advantage that each gene is set to the same (zero) value in basal conditions. We used this representation to identify structures in both the scales and directionalities of temporal responses (Figure 5).

Among genes that show significant induction in all three cell lines, genes in the BG3-c2 cell line respond systematically more rapidly. Expression levels in this cell line also level off more quickly than in S2 and Kc, which show steadily rising expression through all 7 hr.

There are 15 genes that are responsive at 5 hr after induction only in Kc and S2 cells but not in BG3-c2, and these show a strong and consistent expression pattern in the BG3-c2 time course: they are responsive at 1 hr, and then reduce expression level at each time point thereafter. Six of these 15 genes are widespread responsive (p-value < 1e^-10^), including *br* and *Eip71CD*. The rapid (at 1 hr) induction of these genes indicates that BG3-c2 is in a more ecdysone sensitive basal state. Notably, the total responsiveness of these three cell lines differs substantially: the RGC value for BG3-c2 cells is more than twice that of S2 or Kc cells. Taken together, these data indicate that the observed increased responsiveness of BG3-c2 cells at 5 hr may be due to an accelerated ecdysone response relative to other cells.

## Discussion

Leveraging the intrinsic variation in initial state among cell lines, we report the most expansive catalog to date describing the ecdysone response. Our observation that the overall responsiveness of a cell line to ecdysone correlates strongly with EcR mRNA titer indicates that the availability of *EcR* is the primary rate-limiting step in the ecdysone response. Further, we find that *EcR* isoform titer is a powerful predictor of response direction and magnitude for several genes including *br*, a well-studied ecdysone response-regulating gene. Given these observations, it is likely that a substantial fraction of genes induced at 5 hr correspond to direct targets of the EcR/USP heterodimer, though we do not assert that all responses are primary.

We find that genes modulated by ecdysone induction fall broadly into two sets. The smaller set, our “widespread” responders, represents a generalized “ecdysone response unit.” It includes many of the most strongly modulated genes and includes many genes previously identified in genetic and biochemical studies of the ecdysone response. In numbers, the far larger set, our “restricted” responders, represents genes regulated in only one or a few cell lines. The diversity of these responses must reflect varying chromatin, transcription factor, small RNA, and other conditions present in the individual cell lines. Among these, we have identified a substantial number of genes whose responsiveness (positive, negative, or null) varies among the panel of lines. These genes are of special interest because understanding how their behaviors are dictated by cellular state encapsulates the problem we are investigating.

The organization of responsive genes in the genome supports the idea that epigenetic state, including chromatin context, is prominent. Responsive proximal gene pairs preferentially organize on the same strand and a number of bidirectional promoters are found to be under independent control. This is remarkable and indicates that the spatial resolution of ecdysone transduction along the genome is on the order of hundreds of base pairs. Additionally, this responsive gene architecture suggests a role for Pol2-associated chromatin modifications. Furthermore, the widespread and early induction of genes encoding chromatin remodeling factors like *tou* supports the idea that the secondary targets may be determined in part by chromatin remodeling, and that cofactors, in addition to TFs, play essential roles in specifying cellular responses.

Our most significant observation is that the cell line-specific panel of responsive genes can be predicted using the estimated titers of some TFs together with their sequence specific binding motifs. In particular, *br* and *lola* are strong predictors of restricted responses across all 41 cell lines. We estimate that more than a hundred TFs are needed to achieve maximal predictive accuracy, indicating that the interaction of many TFs and cofactors convey divergent gene responses as previously reported (Ihry and Bashirullah 2014).

We count ecdysone-responsive genes by looking for *statistically significant* changes in expression. It is important – even if elementary – to appreciate that a survey of this kind cannot assess *biological significance*. It seems likely that most large changes in expression play important roles, but the majority of responses are small. A twofold threshold is often selected, but is arbitrary. On the one hand, as we have noted above, consistent subthreshold changes in members of important protein complexes are likely to be important even if the individual changes are subthreshold. We must also consider the possibility that some ecdysone responses are incidental in the sense that the receptor binds (directly or indirectly) to a promoter or enhancer sequence that evolved for other purposes. Or, alternatively, as is indicated by the coresponsiveness of proximal genes transcribed on the same strand, local chromatin modifications needed to active a target gene may have auxiliary effects in the genomic neighborhood. A response that is physiologically unnecessary but not harmful will be maintained if the components of the response are needed for other purposes, *e.g.*, in other cell types. Whatever the biological significance (or lack of it) for downstream cytological events of these minor expression changes, understanding the regulatory events that cause them can be expected to shed light on the regulatory syntax we are studying.

The catalog we have produced, along with our initial successes in predicting ecdysone responses, indicate the potential of the system as a model for hormone response. In our view these 41 cell lines, representing 41 individual systems, are governed by regulatory rules that are likely identical, or nearly so, to those of *in vivo Drosophila* cells. They respond to a common signal with diverse responses that must be specified by differing initial states. More detailed studies of these responses, expanded to include small RNAs and coupled with binding site maps for relevant transcription factors, chromatin state profiling, and judiciously designed perturbations, should make it possible to increase the accuracy of prediction and elucidate the underlying biology of hormone response in metazoans.

## Supplementary Material

Supporting Information
