# Supplementary material for: Diverse Hormone Response Networks in 41 Independent Drosophila Cell Lines
Source: G3 (Bethesda). 2016 Jan 12;6(3):683–94. doi: 10.1534/g3.115.023366 (PMC4777130; doi:10.1534/g3.115.023366)
Supplement: Supporting Information [file supp_6_3_683__index.html]

Diverse Hormone Response Networks in 41 Independent Drosophila Cell Lines — Supporting Information 

# Diverse Hormone Response Networks in 41 Independent *Drosophila* Cell Lines

## Supporting Information for Stoiber *et al.*, 2016

**Files in this Data Supplement:**

- Supporting Information - This file contains Figures S1-S5, Tables, S1, S2, S8, S10, S11, S13, and S14, and legends for Tables, S3-S7, S9, and S12. (.pdf, 2,567 KB)
- Figure S4 - Genomic Location of Differentially Expressed Genes. (.pdf, 229 KB)
- Figure S5 - Raw Responsive Proximal Response. (.pdf, 330 KB)
- Table S1 - Additional Properties of Cell Lines in this Study. (.pdf, 36 KB)
- Table S2 - SRA Accession numbers for RNA-seq data reported in this study. (.pdf, 40 KB)
- Table S8 - Robustness of RGC to Thresholds. (.pdf, 12 KB)
- Table S10 - Overlap with Previous Ecdysone Response Studies. (.pdf, 37 KB)
- Table S11 - Divergently Responsive Genes. (.pdf, 29 KB)
- Table S13 - Most Correlated Genes with Fraction of EcR-B1/2 Isoform. (.pdf, 36 KB)
- Figure S1 - Transcription Factor Expression Diversity. (.pdf, 128 KB)
- Table S14 - Most Important Covariates. (.pdf, 35 KB)
- Figure S2 - Gene Level Clustering and Similarity. (.pdf, 99 KB)
- Figure S3 - Cell Line Characteristics Clustered by Restricted Ecdysone Response. (.pdf, 62 KB)
- Table S4 - Raw Exon Counts. (.zip, 7,893 KB)
- Table S5 - Interactive DE Genes Table. (.xlsx, 34,919 KB)
- Table S6 - Identified Ecdysone Responsive Genes with Meta-information. (.xlsx, 553 KB)
- Table S7 - Significantly Enriched GO Terms. (.xlsx, 67 KB)
- Table S9 - Most Correlated Genes with RGC. (.xlsx, 73 KB)
- Table S12 - Significant Exon Level Responses. (.xlsx, 60 KB)
- Table S3 - Raw Gene Counts. (.csv, 4,821 KB)
