# Supplementary material for: Diverse Hormone Response Networks in 41 Independent Drosophila Cell Lines
Source: G3 (Bethesda). 2016 Jan 12;6(3):683–94. doi: 10.1534/g3.115.023366 (PMC4777130; doi:10.1534/g3.115.023366)
Supplement: Supporting Information [file supp_g3.115.023366_023366SI.pdf]

List of Supporting Figures

Figure S1. Transcription Factor Expression Diversity.

Figure S2. Gene Level Clustering and Similarity.

Figure S3. Cell Line Characteristics Clustered by Restricted Ecdysone Response.

Figure S4. Genomic Location of Differentially Expressed Genes.

Figure S5. Raw Responsive Proximal Response.

Figure S6. *Eip75B* Exon Level Ecdysone Response.

Figure S7. *Eip75B* Differential Exon Expression Graphic.

Figure S8. Restricted Response Prediction.

List of Supporting Tables

Table S1. Additional Properties of Cell Lines in this Study

Table S2. SRA Accession numbers for RNA-seq data reported in this study.

Table S3. Raw Gene Counts.

Table S4. Raw Exon Counts.

Table S5. Interactive DE Genes Table.

Table S6. Identified Ecdysone Responsive Genes with Meta-information.

Table S7. Significantly Enriched GO Terms.

Table S8. Robustness of RGC to Thresholds.

Table S9. Most Correlated Genes with RGC.

Table S10. Overlap with Previous Ecdysone Response Studies.

Table S11. Divergently Responsive Genes.

Table S12. Significant Exon Level Responses.

Table S13. Most Correlated Genes with Fraction of *EcR-B1/2* Isoform.

Table S14. Most Important Covariates.

Supporting Figures

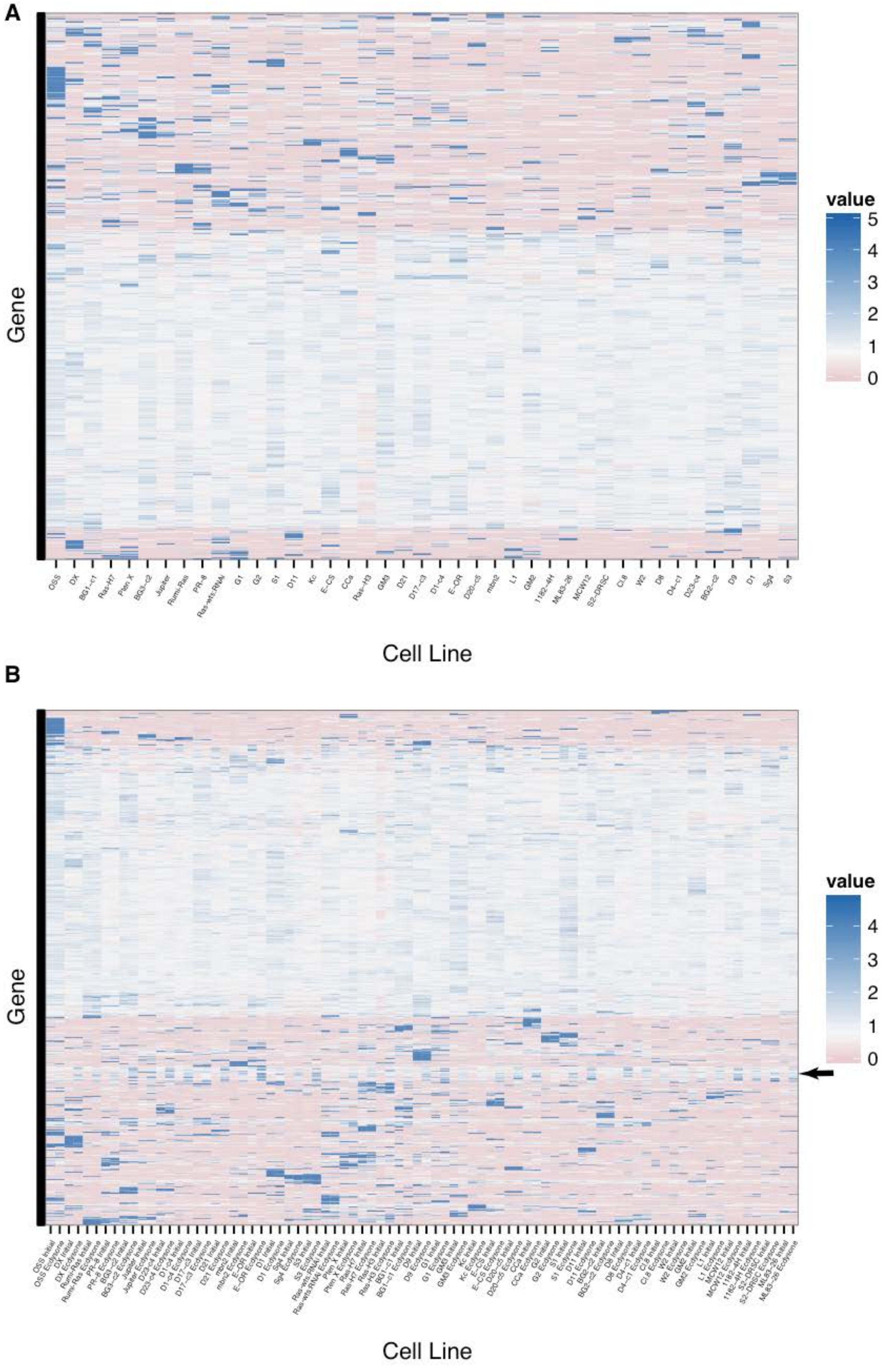

Figure S1. Transcription Factor Expression Diversity. This figure is a heat map representing the expression levels of all transcription factors at the A) zero hour time point and B) the zero and five hour exposure time point. The expression levels are normalized by the mean of expression across all cell lines. Both genes and cell lines are hierarchically clustered based on Euclidian distance. The arrow points the cluster of ecdysone responsive genes as noted by the striped pattern of alternative low and high expression.

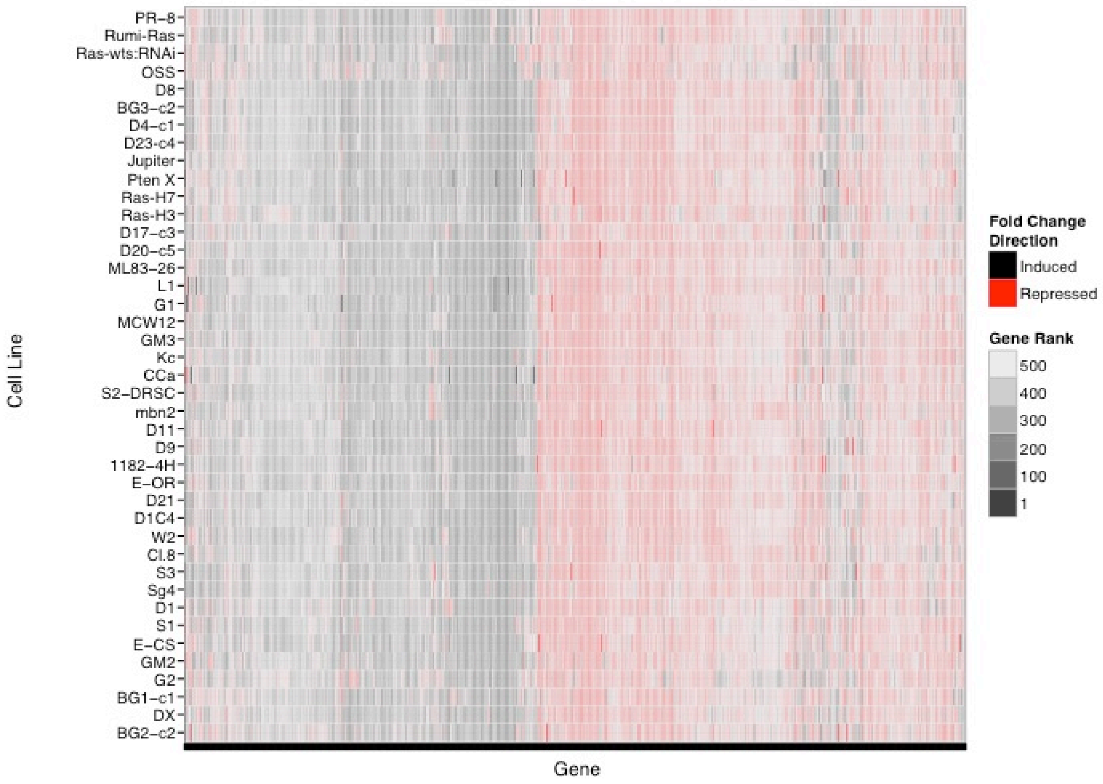

Figure S2. Gene Level Clustering and Similarity. This figure is a heat map representing the response of all genes (along the x axis) that respond significantly in at least two cell lines. The color of each cell represents the direction of response (log fold change greater or less than zero) and the level of transparency represents the rank of the log fold change within each cell line. Both cell lines and genes are clustered (using a hierarchical clustering algorithm).

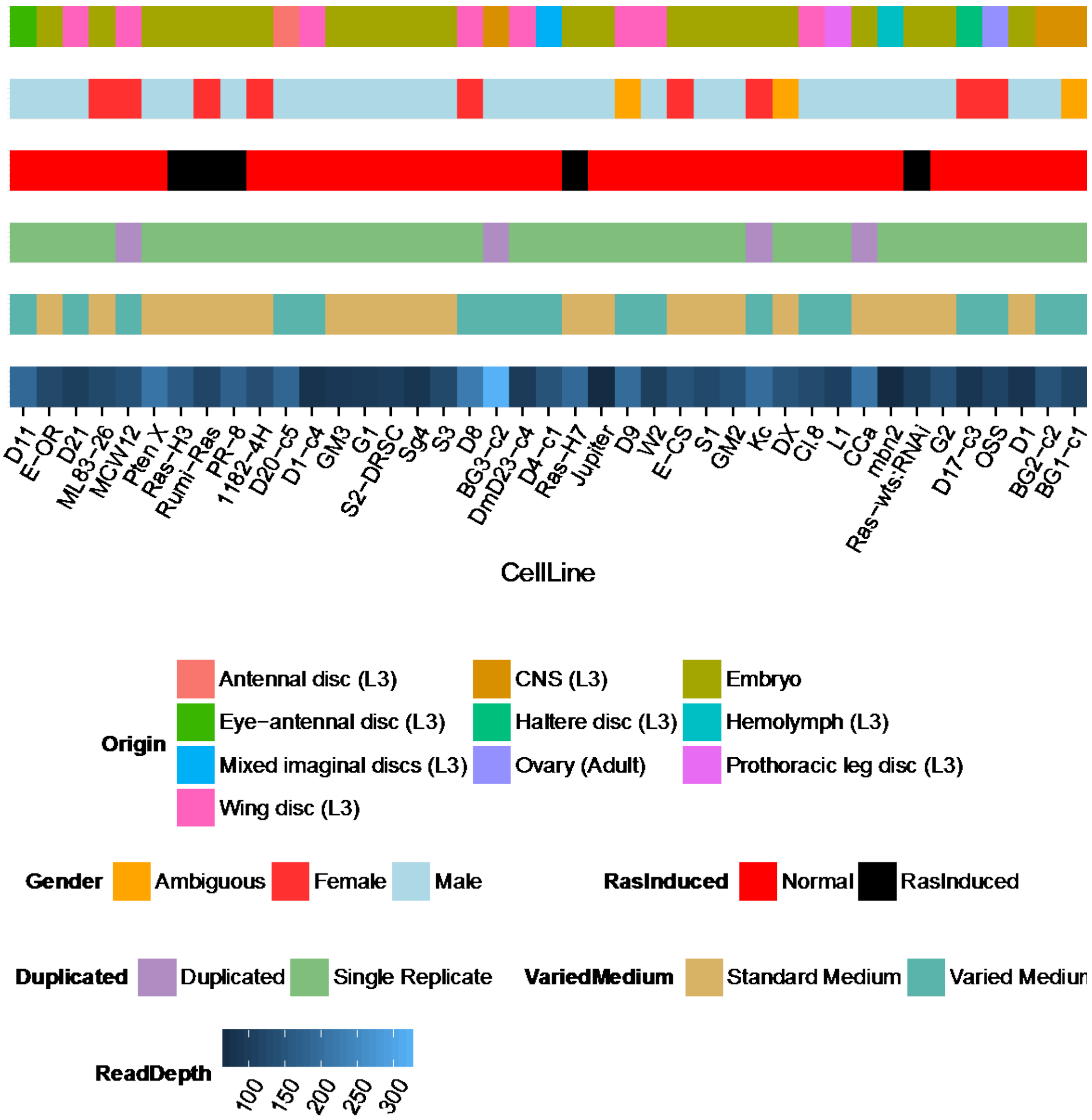

Figure S3. Cell Line Characteristics Clustered by Restricted Ecdysone Response. The cell lines in this figure are clustered in the same order as in Figure 1. Shown in the six panels are the tissue of origin, the gender, the inclusion of constitutively active Ras85D, whether the cell line was measured in biological duplicate, whether a standard media was used and the read depth for all samples from each cell line. The clustering of these covariates with the restricted response is discussed in the main text.

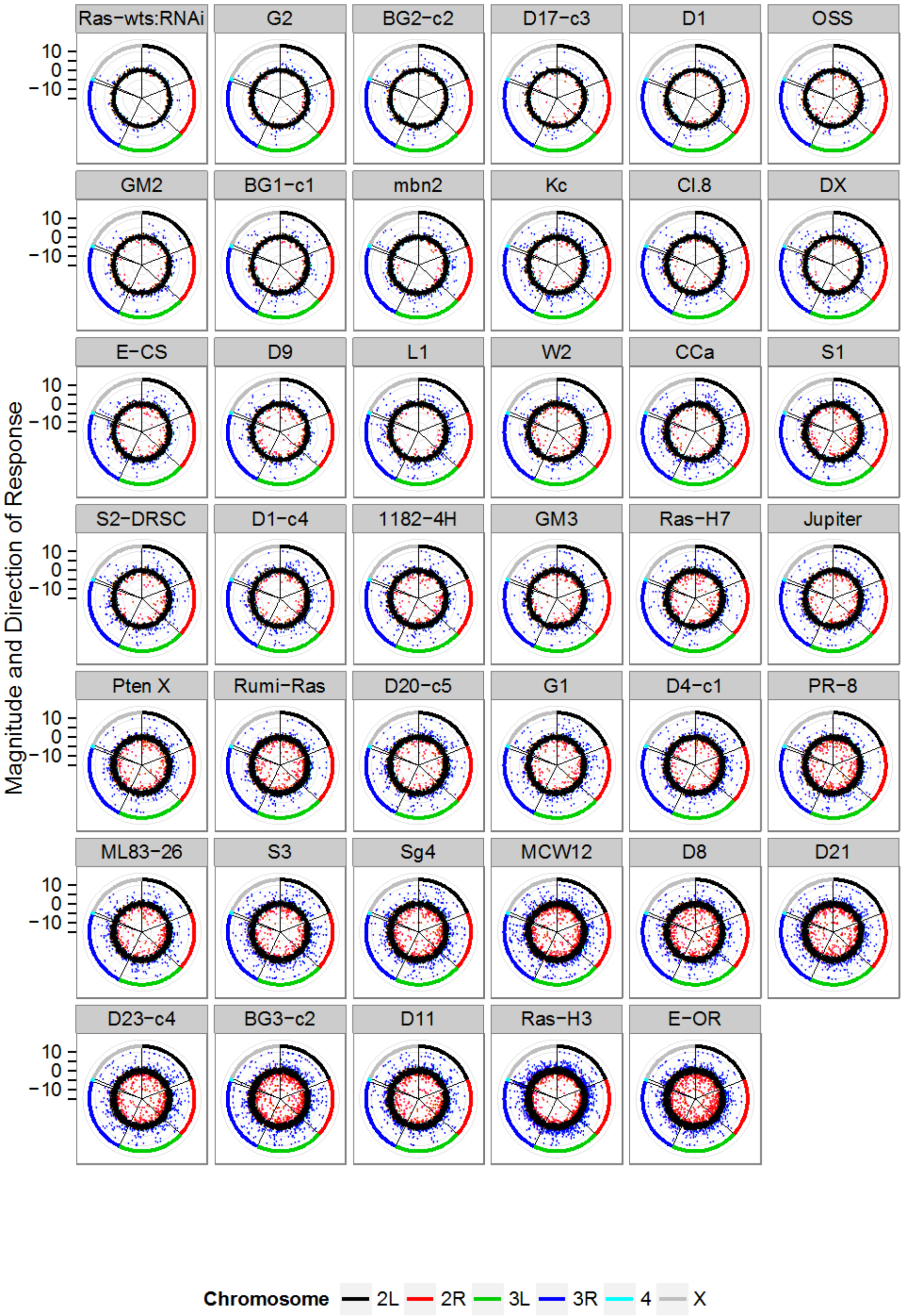

Figure S4. Genomic Location of Differentially Expressed Genes. Each panel represents the ecdysone responsive behavior for a cell line (ordered by the total number of responsive genes). The genomic position is represented on the radial axis. The magnitude and direction of response, as measured by the negative log10 of the differential expression p-value times the direction of response, is represented on the polar axis. Red and blue points are significantly repressed and induced, respectively, in response to ecdysone.

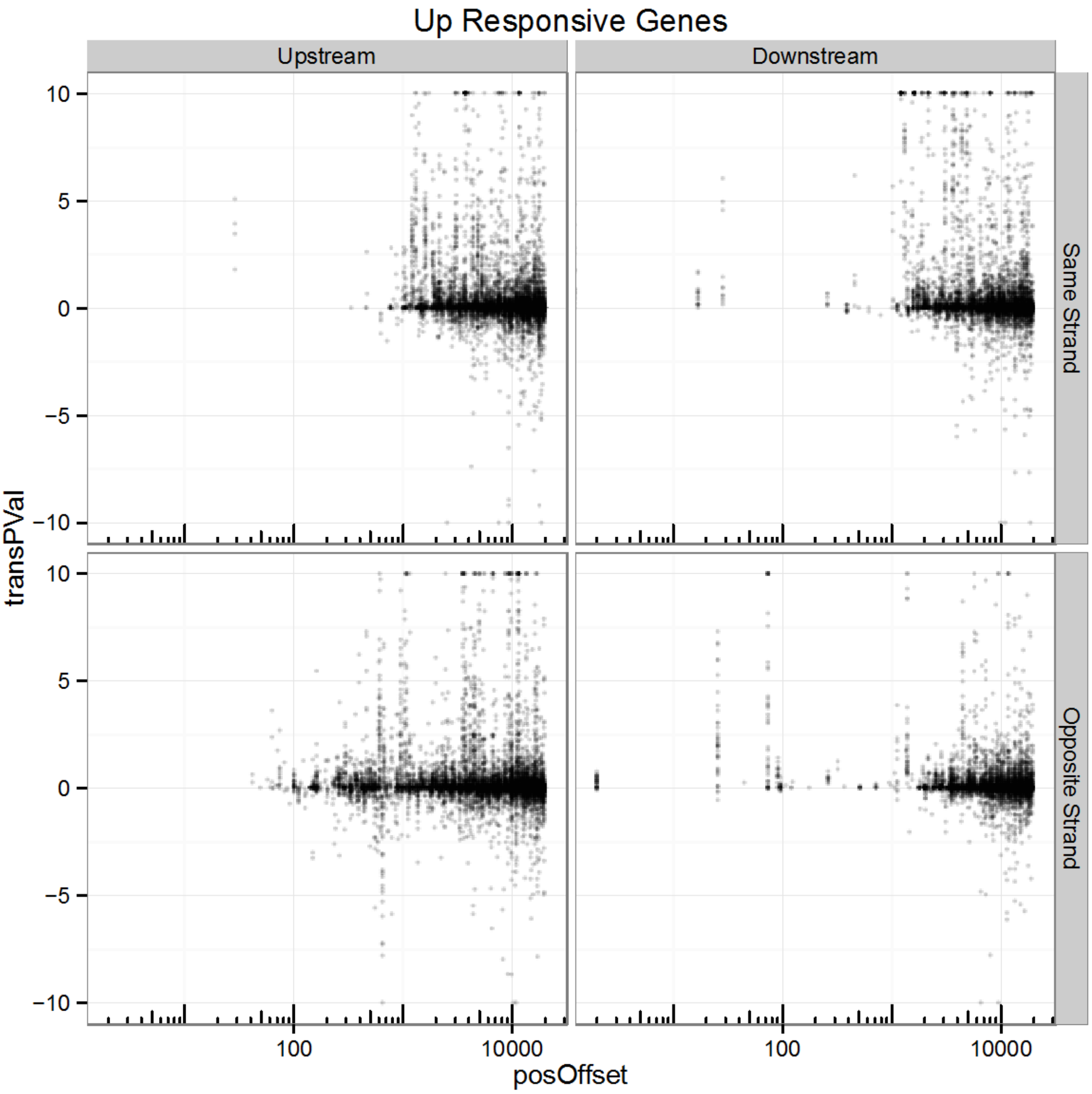

Up Responsive Genes
Upstream
Downstream
Same Strand
Opposite Strand
transPVal
10
5
0
−5
−10
100
10000
posOffset

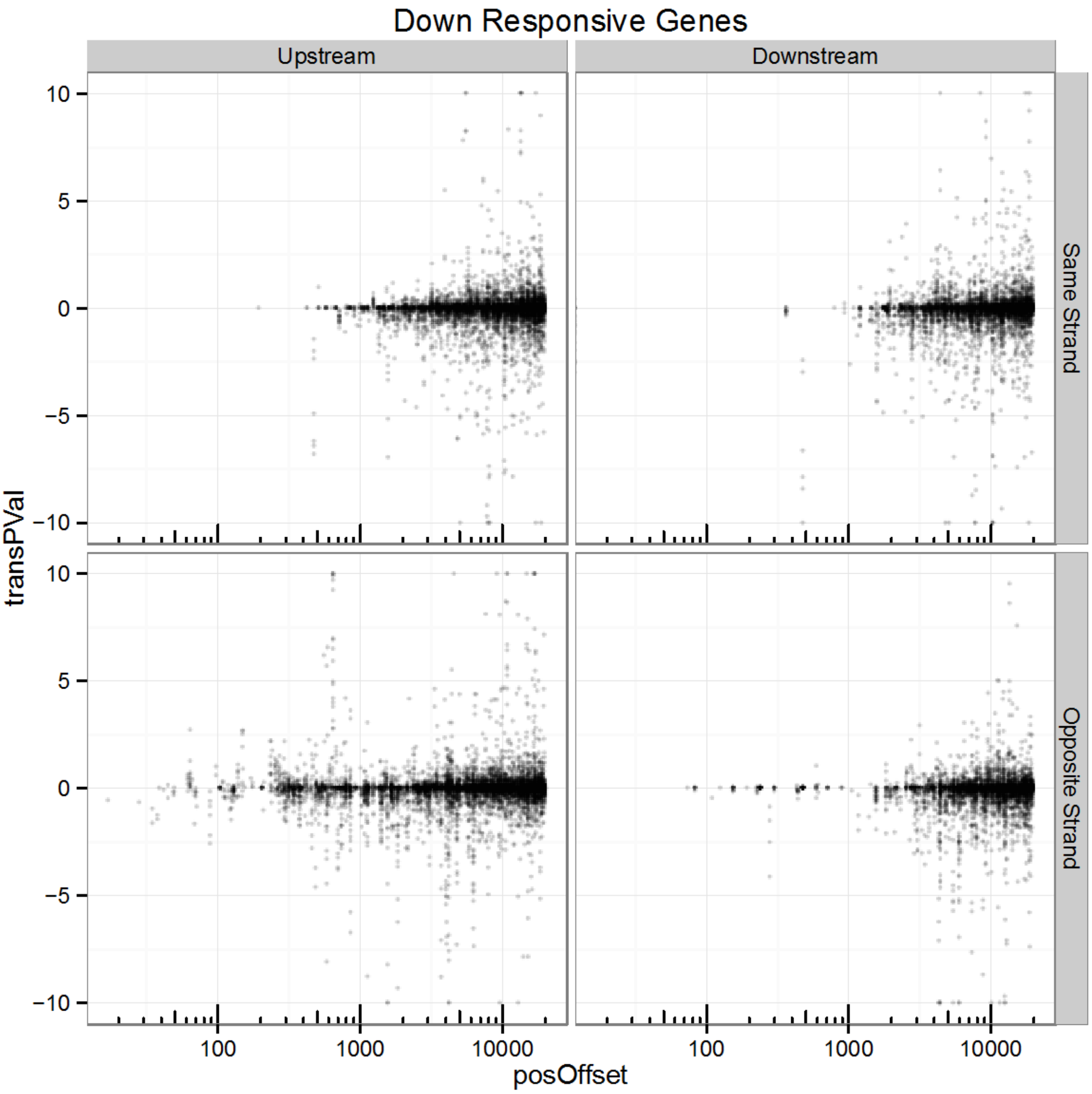

Figure S5. Raw Responsive Proximal Response. Each panel of this figure shows the raw points that produced the smoothed lines found in Figure 3. As in figure three each point represents one gene, cell lines combination for a significantly responsive gene and a proximal gene. Different panels show the responsive behavior of genes proximal to responsive genes segregated by the response direction and the promoter architecture (same/opposite strand and up/down stream promoters).

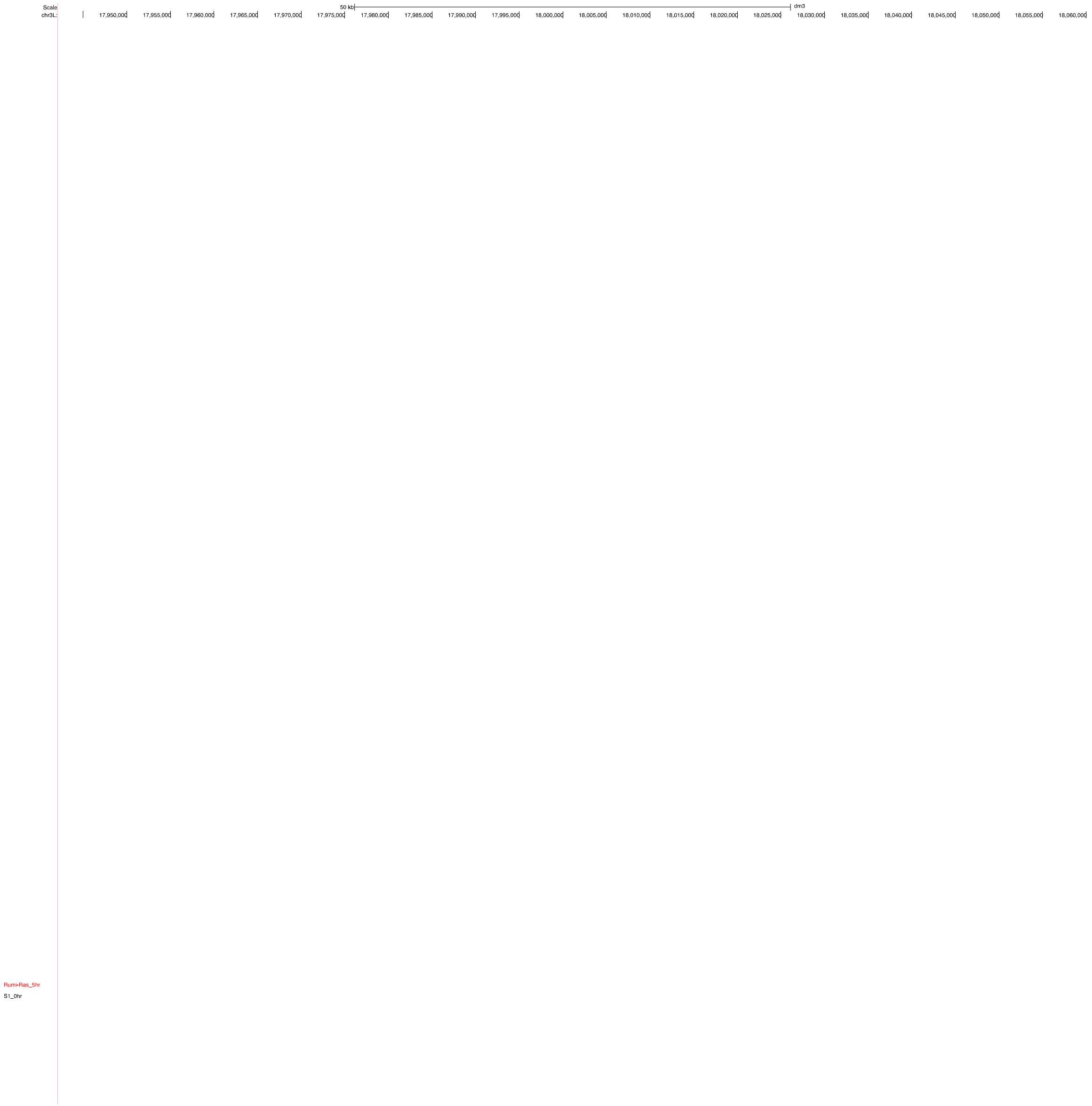

Figure S6. *Eip75B* Exon Level Ecdysone Response. This figure shows a genome browser shot of the *Eip75B* locus showing the global promoter switching event across almost all cell lines.

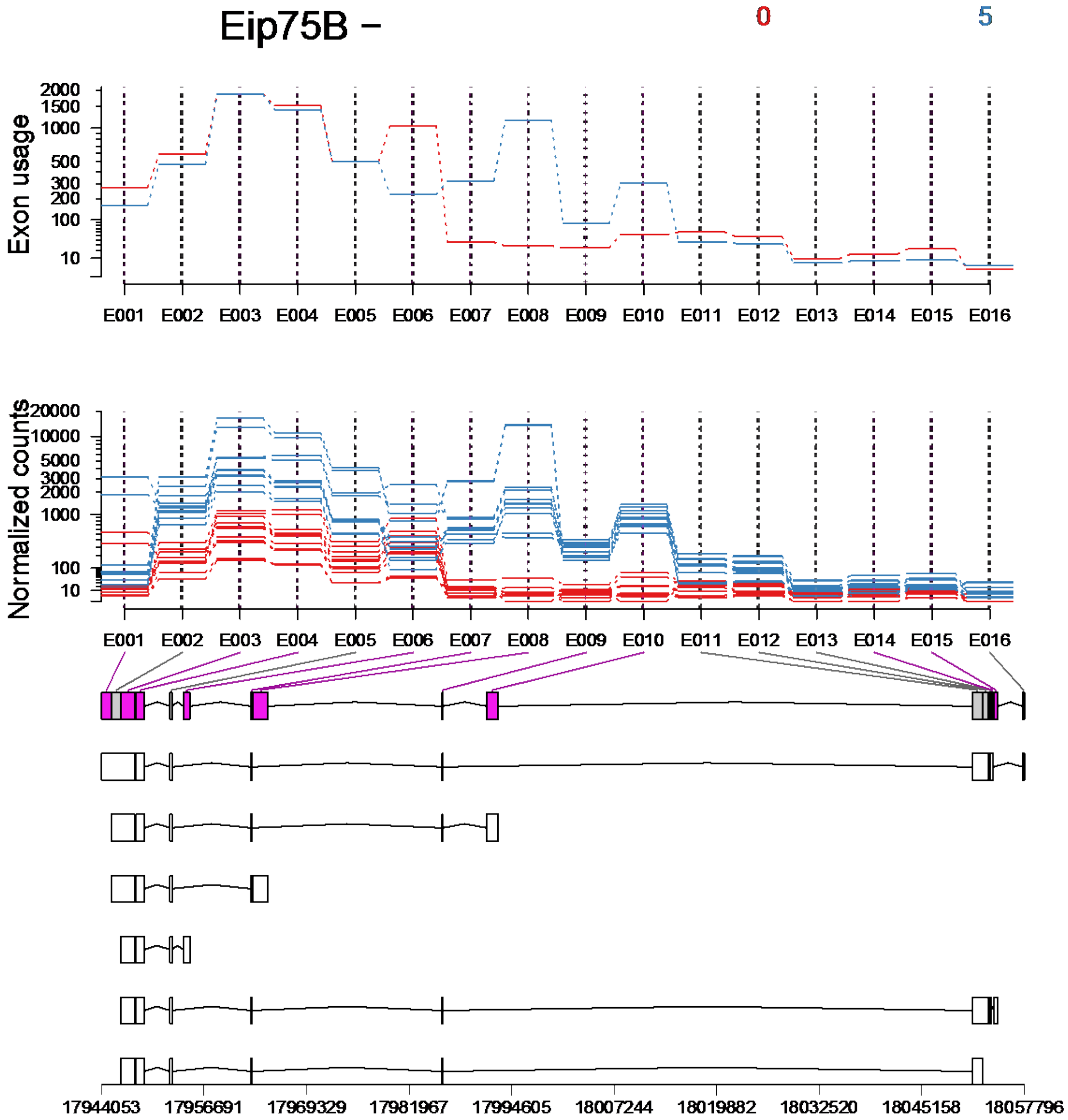

Figure S7. ***Eip75B*** **Differential Exon Expression Graphic**. This figure shows a graphical representation of the promoter-switching event at the *Eip75B* locus. The top panel shows the average exon usage across all cell lines and the second panel shows the exon usage in each cell line individually. The third panel shows the exons identified as differentially expressed in purple as well as the transcript models present in the FlyBase annotation. This figure was produced using the DEXSeq R package.

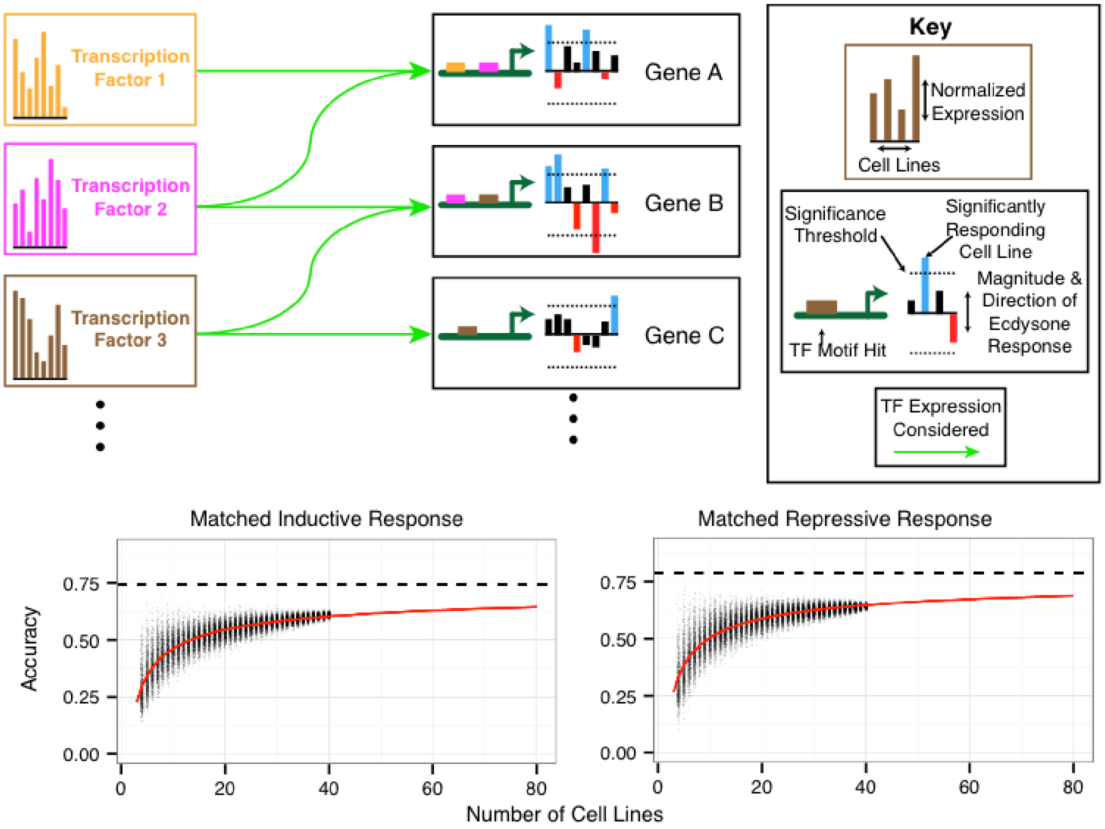

Figure S8. Restricted Response Prediction. **A.** Transcription factor expression levels (left bar plots) combined with known binding motif preference (green arrows indicate promoter motif match) are used to predict whether a cell line is significantly induced (blue bars) or repressed/lowest fold change (red bars) at a locus for genes in the induced restricted set and similarly for the repressed restricted set. Random forests are trained and used to predict response direction on either left out sets of cell lines or left out sets of genes. **B.** Scatterplots showing predictive power when cell lines are left out as a test set for the matched inductive (left panel) and the matched repressive response (right panel). The x-axis indicates the number of cell lines included in the sub-sampled data and the y-axis indicates the average predictive accuracy when each cell line in the sample is left out as test set. Fitted lines in red show average predictive accuracy. Fitted horizontal asymptotes (dashed lines), representing the average predictive accuracy with infinite cell lines available to predict the response of an unseen cell line, are 0.74 for the matched inductive response and 0.79 for the matched repressive response.

Supporting Tables

Table S1. Additional Properties of Cell Lines in this Study

Short name	Formal name	Genotype	Additions to medium	Sex
1182-4H	1182-4H	*mh^-*	--	F(Lee *et al.* 2014)
CCa	CCa	NA	--	M (data in this study)
L1	CME L1	NA	Insulin, fly extract	M (Lee *et al.* 2014)
Cl.8	CME W1 Cl.8+	NA	Insulin, fly extract	M (Lee *et al.* 2014)
W2	CME W2	NA	Insulin, fly extract	M (Lee *et al.* 2014)
D1	D1	NA	--	M (data in this study)
DX	DX	NA	--	Ambiguous (data in this study)
E-CS	E-CS	Wild-type (Canton S)	--	F (data in this study)
E-OR	E-OR	Wild-type (Oregon R)	--	M (data in this study)
G1	G1	NA	--	M (data in this study)
G2	G2	NA	--	M (data in this study)
GM2	GM2	NA	--	M (data in this study)
GM3	GM3	NA	--	M (data in this study)
Jupiter	Jupiter	Jupiter:GFP exon trap	--	M (data in this study)
Kc	Kc167	NA	Serum-free medium CCM-3	F (Lee *et al.* 2014)
mbn2	mbn2	*$mbn2^-$*	--	M (Lee *et al.* 2014)
MCW12	MCW12	NA	Insulin, fly extract	F (data in this study)
ML83-26	ML83-26	Wild-type (Harwich)	--	F (data in this study)
BG1-c1	ML-DmBG1-c1	*y^1 v^1 f^1 mal^{F1}*	Insulin	Ambiguous (data in this study)
BG2-c2	ML-DmBG2-c2	*y^1 v^1 f^1 mal^{F1}*	Insulin	M (data in this study)
BG3-c2	ML-DmBG3-c2	*y^1 v^1 f^1 mal^{F1}*	Insulin	M (Lee *et al.* 2014)
D1-c4	ML-DmD1-c4	*y^1 v^1 f^1 mal^{F1}*	Insulin	M (data in this study)
D11	ML-DmD11	*y^1 v^1 f^1 mal^{F1}*	Insulin	M (data in this study)
D17-c3	ML-DmD17-c3	*y^1 v^1 f^1 mal^{F1}*	Insulin	F (Lee *et al.* 2014)
D20-c5	ML-DmD20-c5	*y^1 v^1 f^1 mal^{F1}*	Insulin	M (Lee *et al.* 2014)
D21	ML-DmD21	*y^1 v^1 f^1 mal^{F1}*	Insulin	M (data in this study)
D23-c4	ML-DmD23-c4	*y^1 v^1 f^1 mal^{F1}*	Insulin	M (data in this study)
D4-c1	ML-DmD4-c1	*y^1 v^1 f^1 mal^{F1}*	Insulin	M (Lee *et al.* 2014)
D8	ML-DmD8	*y^1 v^1 f^1 mal^{F1}*	Insulin	F (Lee *et al.* 2014)
D9	ML-DmD9	*y^1 v^1 f^1 mal^{F1}*	Insulin	Ambiguous (Lee *et al.* 2014)
OSS	OSS	*w^{1118}; P [w^+ hsp70-bam^+] bam^{D86} ry e/bam^{D86} P[ovo-lacZ] P[vas-egfp]*	Insulin, fly extract, glutathione	F (data in this study)
PR-8	PR8	*$Pten^{117}$*; UAS-GFP; P{UAS-Ras85D^{V12}}/ {Act5C-GAL4}/	--	M (data in this study)
Pten X	Pten X	*$Pten^{117}$*	--	M (data in this study)
Ras-wts:RNAi	Ras[V12]; wts[RNAi]	P(Leonardi *et al.*) P{UAS-Ras85D^{V12}} P{UAS-GFP P{UAS-wts-RNAi}/ {Act5C-GAL4}	--	M (data in this study)
Ras-H3	Ras[V12]-H3	UAS-GFP; P{UAS-Ras85D^{V12}}/ {Act5C-GAL4}	--	M (data in this study)
Ras-H7	Ras[V12]-H7	UAS-GFP; P{UAS-Ras85D^{V12}}/ {Act5C-GAL4}/	--	M (data in this study)

Rumi-Ras	rumi[26] Ras[V12]-4	*rumi*^{26} P{Act5C-GAL4}/P{UAS-Ras85D^{V12}}	--	F (data in this study)
S1	S1	Wild-type (Oregon R)	--	M (LEE *et al.* 2014)
S2-DRSC	S2-DRSC	Wild-type (Oregon R)	--	M (LEE *et al.* 2014)
S3	S3	Wild-type (Oregon R)	--	M (LEE *et al.* 2014)
Sg4	Sg4	Wild-type (Oregon R)	--	M (LEE *et al.* 2014)

Listed is additional information for each cell line including genotype, variations to medium and the sex of each cell line along with the associated reference for that determination.

Table S2. SRA Accession numbers for RNA-seq data reported in this study.

**Sample**	**Treatment**	**Treatment Time**	**SRA Accession #**
D1 C	control	----	SRR3038122
D1 E	hormone	5 h	SRR3037437
CME W1 Cl.8+ C	control	----	SRR3038123
CME W1 Cl.8+ E	hormone	5 h	SRR3037943
CME W2 C	control	----	SRR3038127
CME W2 E	hormone	5 h	SRR3037521
CME L1 C	control	----	SRR3038125
CME L1 E	hormone	5 h	SRR3038124
CCa C1	control	---- (sample 1)	SRR3038213
CCa C2	control	---- (sample 2)	SRR3038131
CCa E1	hormone	5 h (sample 1)	SRR3038201
CCa E2	hormone	5 h (sample 2)	SRR3038126
1182-4H C	control	----	SRR3038250
1182-4H E	hormone	5 h	SRR3038240
DX C	control	----	SRR3038251
DX E	hormone	5 h	SRR3038252
E-CS C	control	----	SRR3038283
E-CS E	hormone	5 h	SRR3038284
E-OR C	control	----	SRR3038285
E-OR E	hormone	5 h	SRR3038290
G1 C	control	----	SRR3038293
G1 E	hormone	5 h	SRR3038294
G2 C	control	----	SRR3038296
G2 E	hormone	5 h	SRR3038302
GM2 C	control	----	SRR3038303
GM2 E	hormone	5 h	SRR3038304
GM3 C	control	----	SRR3038306
GM3 E	hormone	5 h	SRR3040020
Jupiter C	control	----	SRR3040046
Jupiter E	hormone	5 h	SRR3040053
Kc167 C-1	control	---- (sample 1)	SRR3040054
Kc167 C-2	control	---- (sample 2)	SRR3040509
Kc167 E1-2	hormone	1 h	SRR3040556

Kc167 E3-2	hormone	3 h	SRR3040557
Kc167 E5-1	hormone	5 h (sample 1)	SRR3040058
Kc167 E5-2	hormone	5 h (sample 2)	SRR3040558
Kc167-E7-2	hormone	7 h	SRR3040559
Kc167 E24-1	hormone	24 h	SRR3040088
mbn2 C	control	----	SRR3040560
mbn2 E	hormone	5 h	SRR3040561
MCW12 C1	control	---- (sample 1)	SRR3040562
MCW12 C2	control	---- (sample 2)	SRR3040674
MCW12 E1	hormone	5 h (sample 1)	SRR3040594
MCW12 E2	hormone	5 h (sample 2)	SRR3040681
ML83-26 C	control	----	SRR3040682
ML83-26 E	hormone	5 h	SRR3041927
ML-DmBG1-c1 C	control	----	SRR3041928
ML-DmBG1-c1 E	hormone	5 h	SRR3041929
ML-DmBG2-c2 C	control	----	SRR3041930
ML-DmBG2-c2 E	hormone	5 h	SRR3041931
ML-DmBG3-c2 C-1	control	---- (sample 1)	SRR3041932
ML-DmBG3-c2 C-2	control	---- (sample 2)	SRR3041935
ML-DmBG3-c2 E1-2	hormone	1 h	SRR3041936
ML-DmBG3-c2 E3-2	hormone	3 h	SRR3041937
ML-DmBG3-c2 E5-1	hormone	5 h (sample 1)	SRR3041933
ML-DmBG3-c2 E5-2	hormone	5 h (sample 2)	SRR3041938
ML-DmBG3-c2 E7-2	hormone	7 h	SRR3041939
ML-DmBG3-c2 E24-1	hormone	24 h	SRR3041934
ML-DmD1-c4 C	control	----	SRR3041940
ML-DmD1-c4 E	hormone	5 h	SRR3041941
ML-DmD11 C	control	----	SRR3041942
ML-DmD11 E	hormone	5 h	SRR3041943
ML-DmD17-c3 C	control	----	SRR3041988
ML-DmD17-c3 E	hormone	5 h	SRR3042072
ML-DmD20-c5 C	control	----	SRR3042157
ML-DmD20-c5 E	hormone	5 h	SRR3042198
ML-DmD21 C	control	----	SRR3042199
ML-DmD21 E	hormone	5 h	SRR3042200
ML-DmD23-c4 C	control	----	SRR3042202
ML-DmD23-c4 E	hormone	5 h	SRR3042203
ML-DmD4-c1 C	control	----	SRR3042204
ML-DmD4-c1 E	hormone	5 h	SRR3042538
ML-DmD8 C	control	----	SRR3042539
ML-DmD8 E	hormone	5 h	SRR3042541
ML-DmD9 C	control	----	SRR3042543
ML-DmD9 E	hormone	5 h	SRR3042546
OSS C	control	----	SRR3042549
OSS E	hormone	5 h	SRR3042550
PR8 C	control	----	SRR3042551

PR8 E	hormone	5 h	SRR3042552
Pten X C	control	----	SRR3042553
Pten X E	hormone	5 h	SRR3042554
Ras[V12]; wts[RNAi] C	control	----	SRR3042555
Ras[V12]; wts[RNAi] E	hormone	5 h	SRR3042556
Ras[V12]-H3 C	control	----	SRR3042557
Ras[V12]-H3 E	hormone	5 h	SRR3042558
Ras[V12]-H7 C	control	----	SRR3042559
Ras[V12]-H7 E	hormone	5 h	SRR3042560
rumi[26] Ras[V12]-4 C	control	----	SRR3042561
rumi[26] Ras[V12}-4 E	hormone	5 h	SRR3042562
S1 C	control	----	SRR3042563
S1 E	hormone	5 h	SRR3042564
S2-DRSC C	control	----	SRR3042565
S2-DRSC E1	hormone	1 h	SRR3042566
S2-DRSC E3	hormone	3 h	SRR3042567
S2-DRSC E5	hormone	5 h	SRR3042568
S2 DRSC E7	hormone	7 h	SRR3042569
S2-DRSC E24	hormone	24 h	SRR3042570
S3 C	control	----	SRR3042571
S3 E	hormone	5 h	SRR3042572
Sg4 C	control	----	SRR3042573
Sg4 E	hormone	5 h	SRR3042574

The SRA accession numbers for each sample reported in this study are presented in this table.

Table S3. Raw Gene Counts. The raw gene counts obtained from all RNA-seq mapped reads are found in this table. The counts presented were calculated using the htseq-count function from the ht-seq package (version 0.6.0) using the *Drosophila* annotation version 5.57. This table can be found in additional supporting documents.

Table S4. Raw Exon Counts. The raw gene counts obtained from mapped reads are found in this table. This table can be found in additional supporting documents.

Table S5. Interactive DE Genes Table. This table shows the p-value and fold change for each gene in each cell line as well as those cell lines interrogated with microarray experiments. The color-coding is dependent upon the editable values in the top left of the spreadsheet (conditional color formatting may not be available for older versions of excel). When these threshold values in these cells are changed all accumulation values as well as charts on the third tab are recalculated.

This table can be found in additional supporting documents. Further descriptions of the interactive functionalities are described on the first tab of the table.

Table S6. Identified Ecdysone Responsive Genes with Meta-information. For each gene identified as significantly responsive in a cell line the Fisher's p-value, calculated p-value, adjusted p-value, and log fold change are listed. This table can be found in additional supporting documents.

Table S7. Significantly Enriched GO Terms. This table shows the most significantly enriched gene ontology terms within the sets of all and weakly induced/repressed genes. This table can be found in additional supporting documents.

Table S8. Robustness of RGC to Thresholds.

		**Fold Change Threshold**						
		**log2(6/5)**	**log2(3/2)**	**log2(5/3)**	**log2(2)**	**log2(5/2)**	**log2(3)**	**log2(4)**
**P-Value Threshold**	**0.001**	0.9862	0.9862	0.9862	0.9889	0.9938	0.9900	0.9714
	**0.0025**	0.9916	0.9916	0.9916	0.9954	0.9954	0.9898	0.9684
	**0.005**	0.9938	0.9938	0.9938	0.9985	0.9955	0.9885	0.9665
	**0.01**	0.9950	0.9950	0.9960	1.0000	0.9949	0.9875	0.9659
	**0.025**	0.9929	0.9929	0.9966	0.9978	0.9922	0.9862	0.9633
	**0.05**	0.9915	0.9934	0.9960	0.9949	0.9890	0.9846	0.9633
	**0.1**	0.9899	0.9923	0.9913	0.9841	0.9834	0.9809	0.9629

For a range of fold change and p-value thresholds the correlation of the RGC with the RGC calculated using the chosen threshold values are shown. Cell values are the Pearson Correlation between the used thresholds and other thresholds on the number of identified hits.

Table S9. Most Correlated Genes with RGC. This table lists the genes that show the largest correlation with the RGC, for which *EcR* shows the strongest correlation, as well as an approximate permutation test p-value. This table can be found in additional supporting documents.

Table S10. Overlap with Previous Ecdysone Response Studies.

Response	Genes	Paper	Tissue /Cell Line	Early/Late	Sequencing Tech.	P-Value	% Hits	# Overlap	# Hits	# Other Hits
Induced	All	Gauhar et al. 2009	Kc	Ealry (1, 3,6)	Microarray	6.86E-82	9.34	87	931	112
Induced	All	Gauhar et al. 2009	Kc	Late (12, 24, 48)	Microarray	2.64E-62	12.03	112	931	267
Induced	All	Gonsalves et al. 2011	Kc	Ealry (4)	Microarray	1.97E-12	2.58	24	931	71
Induced	All	Gonsalves et al. 2011	Salivary	Ealry (4)	Microarray	1.93E-15	3.65	34	931	109
Induced	All	Beckstead et al. 2005	W3 Larvae	Early (6)	Microarray	2.81E-69	16.86	157	931	478
Induced	All	Shlyueva et al. 2014	S2	Late (24)	RNA-seq	4.33E-107	32.22	300	931	1159
Induced	Widespread	Gauhar et al. 2009	Kc	Ealry (1, 3,6)	Microarray	5.04E-44	41.18	28	68	112
Induced	Widespread	Gauhar et al. 2009	Kc	Late (12, 24, 48)	Microarray	7.24E-34	42.65	29	68	267
Induced	Widespread	Gonsalves et al. 2011	Kc	Ealry (4)	Microarray	1.55E-12	13.24	9	68	71
Induced	Widespread	Gonsalves et al. 2011	Salivary	Ealry (4)	Microarray	2.88E-09	11.76	8	68	109
Induced	Widespread	Beckstead et al. 2005	W3 Larvae	Early (6)	Microarray	1.39E-27	44.12	30	68	478
Induced	Widespread	Shlyueva et al. 2014	S2	Late (24)	RNA-seq	5.41E-38	70.59	48	68	1159
Induced	Restricted	Gauhar et al. 2009	Kc	Ealry (1, 3,6)	Microarray	1.14E-42	6.84	59	863	112
Induced	Restricted	Gauhar et al. 2009	Kc	Late (12, 24, 48)	Microarray	3.18E-37	9.62	83	863	267
Induced	Restricted	Gonsalves et al. 2011	Kc	Ealry (4)	Microarray	5.94E-06	1.74	15	863	71
Induced	Restricted	Gonsalves et al. 2011	Salivary	Ealry (4)	Microarray	4.30E-10	3.01	26	863	109
Induced	Restricted	Beckstead et al. 2005	W3 Larvae	Early (6)	Microarray	4.77E-48	14.72	127	863	478
Induced	Restricted	Shlyueva et al. 2014	S2	Late (24)	RNA-seq	3.05E-78	29.20	252	863	1159
Repressed	All	Gauhar et al. 2009	Kc	Ealry (1, 3,6)	Microarray	4.62E-17	3.14	25	797	60
Repressed	All	Gauhar et al. 2009	Kc	Late (12, 24, 48)	Microarray	1.43E-15	9.66	77	797	510
Repressed	All	Gonsalves et al. 2011	Kc	Ealry (4)	Microarray	3.18E-09	2.38	19	797	73
Repressed	All	Beckstead et al. 2005	W3 Larvae	Early (6)	Microarray	0.000236953	1.76	14	797	92
Repressed	All	Shlyueva et al. 2014	S2 Late	Late (24)	RNA-seq	5.86E-57	28.86	230	797	1347
Repressed	Widespread	Gauhar et al. 2009	Kc	Ealry (1, 3,6)	Microarray	6.35E-11	18.75	6	32	60
Repressed	Widespread	Gauhar et al. 2009	Kc	Late (12, 24, 48)	Microarray	1.58E-06	25.00	8	32	510
Repressed	Widespread	Gonsalves et al. 2011	Kc	Ealry (4)	Microarray	2.32E-05	9.38	3	32	73
Repressed	Widespread	Beckstead et al. 2005	W3 Larvae	Early (6)	Microarray	0.019172858	3.12	1	32	92
Repressed	Widespread	Shlyueva et al. 2014	S2 Late	Late (24)	RNA-seq	8.26E-10	50.00	16	32	1347

Repressed	Restricted	Gauhar et al. 2009	Kc	Ealry (1, 3,6)	Microarray	3.04E-11	2.48	19	765	60
Repressed	Restricted	Gauhar et al. 2009	Kc	Late (12, 24, 48)	Microarray	1.32E-12	9.02	69	765	510
Repressed	Restricted	Gonsalves et al. 2011	Kc	Ealry (4)	Microarray	3.52E-07	2.09	16	765	73
Repressed	Restricted	Beckstead et al. 2005	W3 Larvae	Early (6)	Microarray	0.000517665	1.7	13	765	92
Repressed	Restricted	Shlyueva et al. 2014	S2 Late	Late (24)	RNA-seq	2.42E-50	27.97	214	765	1347

The overlap of widespread, restricted and all responsive genes with previous studies are shown along with the hypergeometric p-value for this overlap.

Table S11. Divergently Responsive Genes.

Gene Name	Number of Induced Cell Lines	Number of Repressed Cell Lines
Oatp74D	5	16
CG9932	5	6
alpha-Est9	10	4
CG13252	4	10
Mal-A5	4	3
sda	3	9
SKIP	3	9
Mct1	9	2
CG32425	6	2
Toll-7	4	2
CG9743	2	21
Argk	2	8
Timp	2	7
CG3036	2	6
bmm	2	5
Pino	2	3
Ama	36	1
CG11509	26	1
CG9416	26	1
br	23	1
eater	20	1
Cyp12a4	13	1
Ugt58Fa	7	1
CG17278	4	1
CrebA	4	1
Apoltp	3	1
bnl	3	1
Cyp9f2	2	1
Dyrk2	2	1
kon	2	1
sick	2	1

Swim	2	1
Cyp6a13	1	17
CG31710	1	14
CG7255	1	14
GstT4	1	14
Fas3	1	13
Ndae1	1	13
babos	1	12
yin	1	11
CG15695	1	10
CG31522	1	10
Lrch	1	10
CG17646	1	9
rau	1	9
CG43795	1	7
Rcd2	1	7
CG3376	1	6
Ih	1	6
CG6357	1	5
CG9521	1	5
pgant2	1	5
CG10527	1	4
CG14995	1	4
CG18446	1	4
nrv3	1	4
ths	1	4
CG42240	1	3
Drat	1	3
Imp	1	3
tutl	1	3
CBP	1	2
CG31219	1	2
CG9981	1	2
PH4alphaEFB	1	2
Prestin	1	2
btl	1	1
CG13868	1	1
CG17124	1	1
CG17150	1	1
CG17834	1	1
CG34232	1	1
CG43164	1	1
CR44526	1	1
Cyp6w1	1	1
E2f	1	1
ena	1	1
Idgf3	1	1
Mbs	1	1

Plod	1	1
stai	1	1
sty	1	1
Traf4	1	1

This table shows the genes that respond significantly inductively in some cell line(s) and significantly repressively in other cell line(s).

Table S12. Significant Exon Level Responses. Those exons identified as having significant differential exon usage are shown along with the p-value and fold change associated with the exon usage. This table can be found in additional supporting documents.

Table S13. Most Correlated Genes with Fraction of *EcR-B1/2* Isoform.

Gene	Correlation with Fraction of Short EcR Isofrom	Permutation Test P-Value	Adjusted P-Value
glec	-0.644928268	0	0
sqz	0.660407469	4.00E-06	0.012388333
CG5059	0.59833374	8.00E-06	0.012388333
Eip55E	0.571066986	8.00E-06	0.012388333
br	0.633163455	1.20E-05	0.012388333
CG5335	0.634343237	1.40E-05	0.012388333
CG14440	0.599282244	1.40E-05	0.012388333
Xbp1	-0.586784569	1.50E-05	0.012388333
CG4825	0.560477019	1.50E-05	0.012388333
CG5482	0.581179456	2.00E-05	0.014294231
mlt	0.483261053	2.30E-05	0.014294231
LIMK1	0.602181835	2.50E-05	0.014294231
cdi	-0.617214213	2.50E-05	0.014294231
CG34330	-0.581866181	3.50E-05	0.0185825
CG5359	0.5953757	4.00E-05	0.019238353
Alh	0.577959631	4.30E-05	0.019238353
loj	-0.57636296	4.40E-05	0.019238353
CG2865	0.496826963	5.70E-05	0.022690211
osp	-0.574710543	5.80E-05	0.022690211
Tsp42Ee	-0.598284087	6.30E-05	0.02341395
CG31370	0.576136587	8.40E-05	0.029219379
CG1418	-0.550475603	9.10E-05	0.029219379
tws	0.495246729	9.10E-05	0.029219379
Glycogenin	0.55734144	9.90E-05	0.029219379
Syp	0.54993558	0.00011	0.029219379
CG32428	0.594831217	0.000111	0.029219379
CG3408	0.551354562	0.000113	0.029219379
SdhA	-0.529640291	0.000114	0.029219379
Klp64D	-0.528184046	0.000114	0.029219379

CG2247	0.534616716	0.000122	0.030227533
CG9005	0.544531433	0.00014	0.033568387
cnc	0.526806645	0.000147	0.033885735
Hmg-2	-0.534014497	0.000151	0.033885735
Rel	-0.542420057	0.000155	0.033885735
CG13624	0.556401565	0.000174	0.036952629
Cbp80	0.499257521	0.000196	0.039575703
CG3587	0.539226318	0.000197	0.039575703
CycE	0.53696766	0.000206	0.040173595
Sans	-0.478439908	0.000218	0.040173595
RabX1	0.559355235	0.000221	0.040173595
mnb	0.50065999	0.000222	0.040173595
Pbgs	-0.51176193	0.000227	0.040173595
Su(dx)	-0.497795752	0.000251	0.043387977
CG9323	-0.541485883	0.000276	0.046625182
Itgbetanu	0.520438966	0.000288	0.0475712
CG11739	-0.529675673	0.000314	0.050133213
CG17036	0.504040971	0.000317	0.050133213
CG9149	-0.504389999	0.00033	0.050719294
SCOT	-0.486500621	0.000342	0.050719294
ihog	0.51369445	0.000347	0.050719294
CG14971	-0.520464111	0.000348	0.050719294
ena	0.493287985	0.000377	0.052534932
tai	0.472951129	0.000382	0.052534932
CG3781	0.503481214	0.00039	0.052534932
CG43658	0.467565063	0.000397	0.052534932
E2f	0.502944659	0.000404	0.052534932
rho	-0.521886478	0.000406	0.052534932
CG12560	0.494852351	0.000415	0.052534932
Cog7	-0.505347582	0.000417	0.052534932
CG7943	-0.493436474	0.000453	0.05611915
CG8858	0.473405421	0.000497	0.060063435
jet	-0.478195889	0.000501	0.060063435
CG17029	0.474493467	0.000554	0.063968848
rhea	0.496542017	0.000562	0.063968848
peb	0.498685766	0.000564	0.063968848
qkr58E-1	0.476232951	0.000568	0.063968848
CG15170	0.471215173	0.000577	0.064012552
CG14906	-0.485501257	0.000598	0.065366676
CG9743	-0.51104703	0.000611	0.065819754
CG7484	-0.488968967	0.000657	0.068924182
Lasp	0.488775298	0.000674	0.068924182
Tapdelta	-0.478834328	0.000682	0.068924182
CG9590	-0.472750658	0.000691	0.068924182
CG6330	-0.469422457	0.000699	0.068924182
RnrS	0.485264857	0.000701	0.068924182
CG9705	0.49725942	0.00071	0.068924182
CG7556	-0.496916291	0.000714	0.068924182

CG3703	0.502312479	0.000732	0.069155127
GlcAT-P	-0.510481353	0.000735	0.069155127
CG7565	-0.469127833	0.000756	0.07024185
Prestin	0.488607675	0.000823	0.075522951
CG31777	-0.485442491	0.000883	0.079524145
garz	-0.49225042	0.000888	0.079524145
CG14907	-0.476211731	0.000922	0.081586024
dmGlut	0.487515735	0.000977	0.085435776
mdlc	0.485664353	0.001023	0.088418128
Sec24CD	-0.481288453	0.001051	0.089794057
CG5001	-0.477730496	0.001098	0.092286124
CG32262	-0.467627802	0.001105	0.092286124
Mal-B2	-0.497905227	0.00113	0.093325444
Cyp28d1	-0.475526108	0.00128	0.104552088
CG15111	-0.468546288	0.001313	0.106081837
CG7265	0.47074843	0.001413	0.112933645
CG6479	0.467233347	0.001455	0.115053351
Letm1	-0.472584042	0.001494	0.116893705
dre4	0.476710975	0.001587	0.122876781
RhoL	-0.472579311	0.001747	0.132959684
CG34376	0.497281292	0.001753	0.132959684
bur	0.471887383	0.001822	0.136797232
CG31098	0.472187578	0.002067	0.15364011

The genes with normalized expression showing the largest correlation with the fraction of the *ErC-B1/2* isoform are shown in this table.

Table S14. Most Important Covariates.

Gene	Correlation with Fraction of Short EcR Isofrom	Permutation Test P-Value	Adjusted P-Value
glec	-0.644928268	0	0
sqz	0.660407469	4.00E-06	0.012388333
CG5059	0.59833374	8.00E-06	0.012388333
Eip55E	0.571066986	8.00E-06	0.012388333
br	0.633163455	1.20E-05	0.012388333
CG5335	0.634343237	1.40E-05	0.012388333
CG14440	0.599282244	1.40E-05	0.012388333
Xbp1	-0.586784569	1.50E-05	0.012388333
CG4825	0.560477019	1.50E-05	0.012388333
CG5482	0.581179456	2.00E-05	0.014294231
mlt	0.483261053	2.30E-05	0.014294231
LIMK1	0.602181835	2.50E-05	0.014294231
cdi	-0.617214213	2.50E-05	0.014294231
CG34330	-0.581866181	3.50E-05	0.0185825
CG5359	0.5953757	4.00E-05	0.019238353
Alh	0.577959631	4.30E-05	0.019238353

loj	-0.57636296	4.40E-05	0.019238353
CG2865	0.496826963	5.70E-05	0.022690211
osp	-0.574710543	5.80E-05	0.022690211
Tsp42Ee	-0.598284087	6.30E-05	0.02341395
CG31370	0.576136587	8.40E-05	0.029219379
CG1418	-0.550475603	9.10E-05	0.029219379
tws	0.495246729	9.10E-05	0.029219379
Glycogenin	0.55734144	9.90E-05	0.029219379
Syp	0.54993558	0.00011	0.029219379
CG32428	0.594831217	0.000111	0.029219379
CG3408	0.551354562	0.000113	0.029219379
SdhA	-0.529640291	0.000114	0.029219379
Klp64D	-0.528184046	0.000114	0.029219379
CG2247	0.534616716	0.000122	0.030227533
CG9005	0.544531433	0.00014	0.033568387
cnc	0.526806645	0.000147	0.033885735
Hmg-2	-0.534014497	0.000151	0.033885735
Rel	-0.542420057	0.000155	0.033885735
CG13624	0.556401565	0.000174	0.036952629
Cbp80	0.499257521	0.000196	0.039575703
CG3587	0.539226318	0.000197	0.039575703
CycE	0.53696766	0.000206	0.040173595
Sans	-0.478439908	0.000218	0.040173595
RabX1	0.559355235	0.000221	0.040173595
mnb	0.50065999	0.000222	0.040173595
Pbgs	-0.51176193	0.000227	0.040173595
Su(dx)	-0.497795752	0.000251	0.043387977
CG9323	-0.541485883	0.000276	0.046625182
Itgbetanu	0.520438966	0.000288	0.0475712
CG11739	-0.529675673	0.000314	0.050133213
CG17036	0.504040971	0.000317	0.050133213
CG9149	-0.504389999	0.00033	0.050719294
SCOT	-0.486500621	0.000342	0.050719294
ihog	0.51369445	0.000347	0.050719294
CG14971	-0.520464111	0.000348	0.050719294
ena	0.493287985	0.000377	0.052534932
tai	0.472951129	0.000382	0.052534932
CG3781	0.503481214	0.00039	0.052534932
CG43658	0.467565063	0.000397	0.052534932
E2f	0.502944659	0.000404	0.052534932
rho	-0.521886478	0.000406	0.052534932
CG12560	0.494852351	0.000415	0.052534932
Cog7	-0.505347582	0.000417	0.052534932
CG7943	-0.493436474	0.000453	0.05611915
CG8858	0.473405421	0.000497	0.060063435
jet	-0.478195889	0.000501	0.060063435
CG17029	0.474493467	0.000554	0.063968848
rhea	0.496542017	0.000562	0.063968848

peb	0.498685766	0.000564	0.063968848
qkr58E-1	0.476232951	0.000568	0.063968848
CG15170	0.471215173	0.000577	0.064012552
CG14906	-0.485501257	0.000598	0.065366676
CG9743	-0.51104703	0.000611	0.065819754
CG7484	-0.488968967	0.000657	0.068924182
Lasp	0.488775298	0.000674	0.068924182
Tapdelta	-0.478834328	0.000682	0.068924182
CG9590	-0.472750658	0.000691	0.068924182
CG6330	-0.469422457	0.000699	0.068924182
RnrS	0.485264857	0.000701	0.068924182
CG9705	0.49725942	0.00071	0.068924182
CG7556	-0.496916291	0.000714	0.068924182
CG3703	0.502312479	0.000732	0.069155127
GlcAT-P	-0.510481353	0.000735	0.069155127
CG7565	-0.469127833	0.000756	0.07024185
Prestin	0.488607675	0.000823	0.075522951
CG31777	-0.485442491	0.000883	0.079524145
garz	-0.49225042	0.000888	0.079524145
CG14907	-0.476211731	0.000922	0.081586024
dmGlut	0.487515735	0.000977	0.085435776
mdlc	0.485664353	0.001023	0.088418128
Sec24CD	-0.481288453	0.001051	0.089794057
CG5001	-0.477730496	0.001098	0.092286124
CG32262	-0.467627802	0.001105	0.092286124
Mal-B2	-0.497905227	0.00113	0.093325444
Cyp28d1	-0.475526108	0.00128	0.104552088
CG15111	-0.468546288	0.001313	0.106081837
CG7265	0.47074843	0.001413	0.112933645
CG6479	0.467233347	0.001455	0.115053351
Letm1	-0.472584042	0.001494	0.116893705
dre4	0.476710975	0.001587	0.122876781
RhoL	-0.472579311	0.001747	0.132959684
CG34376	0.497281292	0.001753	0.132959684
bur	0.471887383	0.001822	0.136797232
CG31098	0.472187578	0.002067	0.15364011

This table includes the transcription factor importance values as well as importance values relative to the 20th most important variable from the model fitting procedure for the top most important variables.
