# Supplementary material for: Diverse Hormone Response Networks in 41 Independent Drosophila Cell Lines
Source: G3 (Bethesda). 2016 Jan 12;6(3):683–94. doi: 10.1534/g3.115.023366 (PMC4777130; doi:10.1534/g3.115.023366)
Supplement: Supporting Information [file supp_g3.115.023366_FigureS4.pdf]

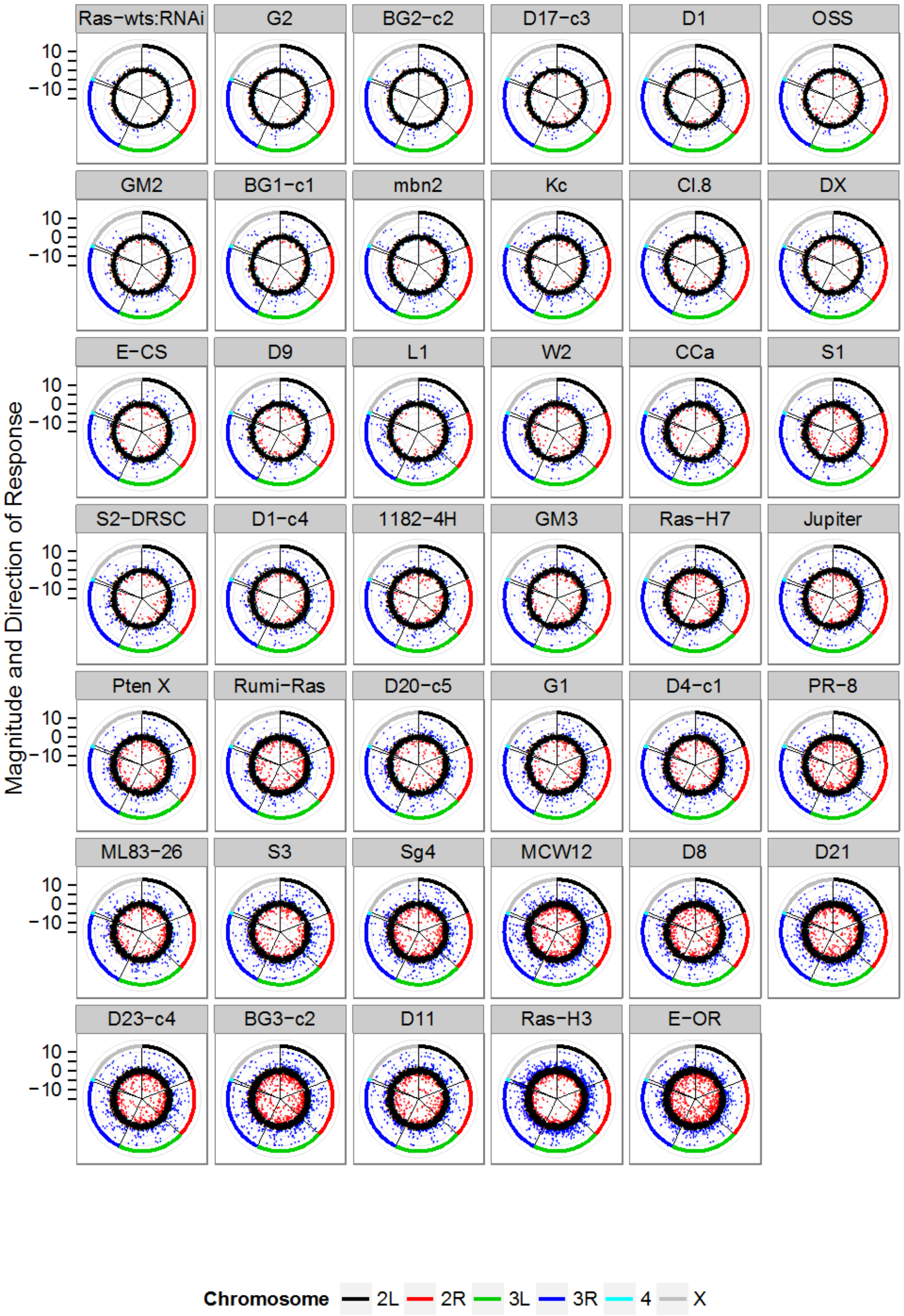

Figure S4. Genomic Location of Differentially Expressed Genes. Each panel represents the ecdysone responsive behavior for a cell line (ordered by the total number of responsive genes). The genomic position is represented on the radial axis. The magnitude and direction of response, as measured by the negative log10 of the differential expression p-value times the direction of response, is represented on the polar axis. Red and blue points are significantly repressed and induced, respectively, in response to ecdysone.
