# Supplementary material for: Diverse Hormone Response Networks in 41 Independent Drosophila Cell Lines
Source: G3 (Bethesda). 2016 Jan 12;6(3):683–94. doi: 10.1534/g3.115.023366 (PMC4777130; doi:10.1534/g3.115.023366)
Supplement: Supporting Information [file supp_g3.115.023366_FigureS5.pdf]

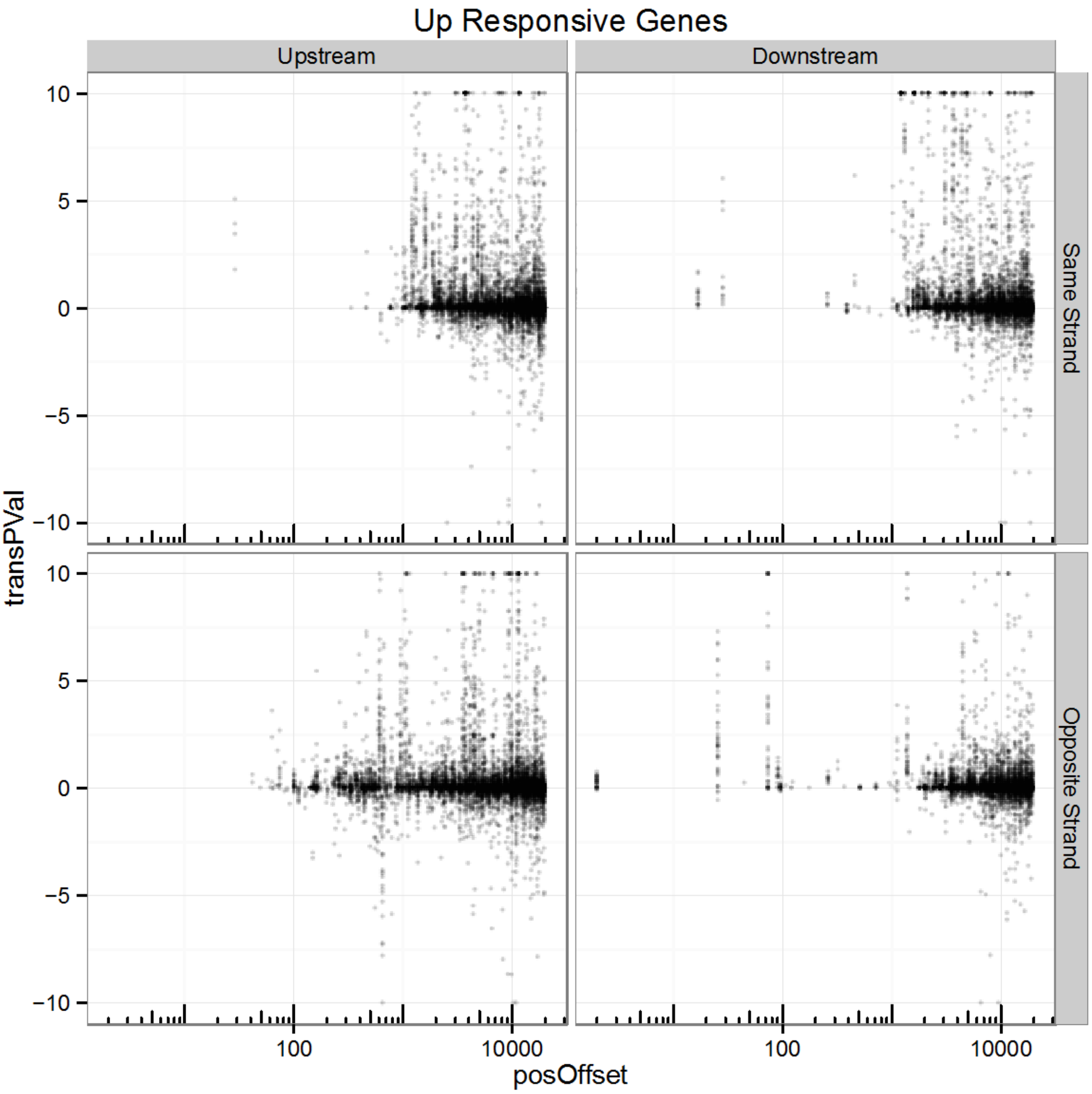
Up Responsive Genes
Upstream
Downstream
Same Strand
Opposite Strand
transPVal
posOffset
10
5
0
−5
−10
100
10000

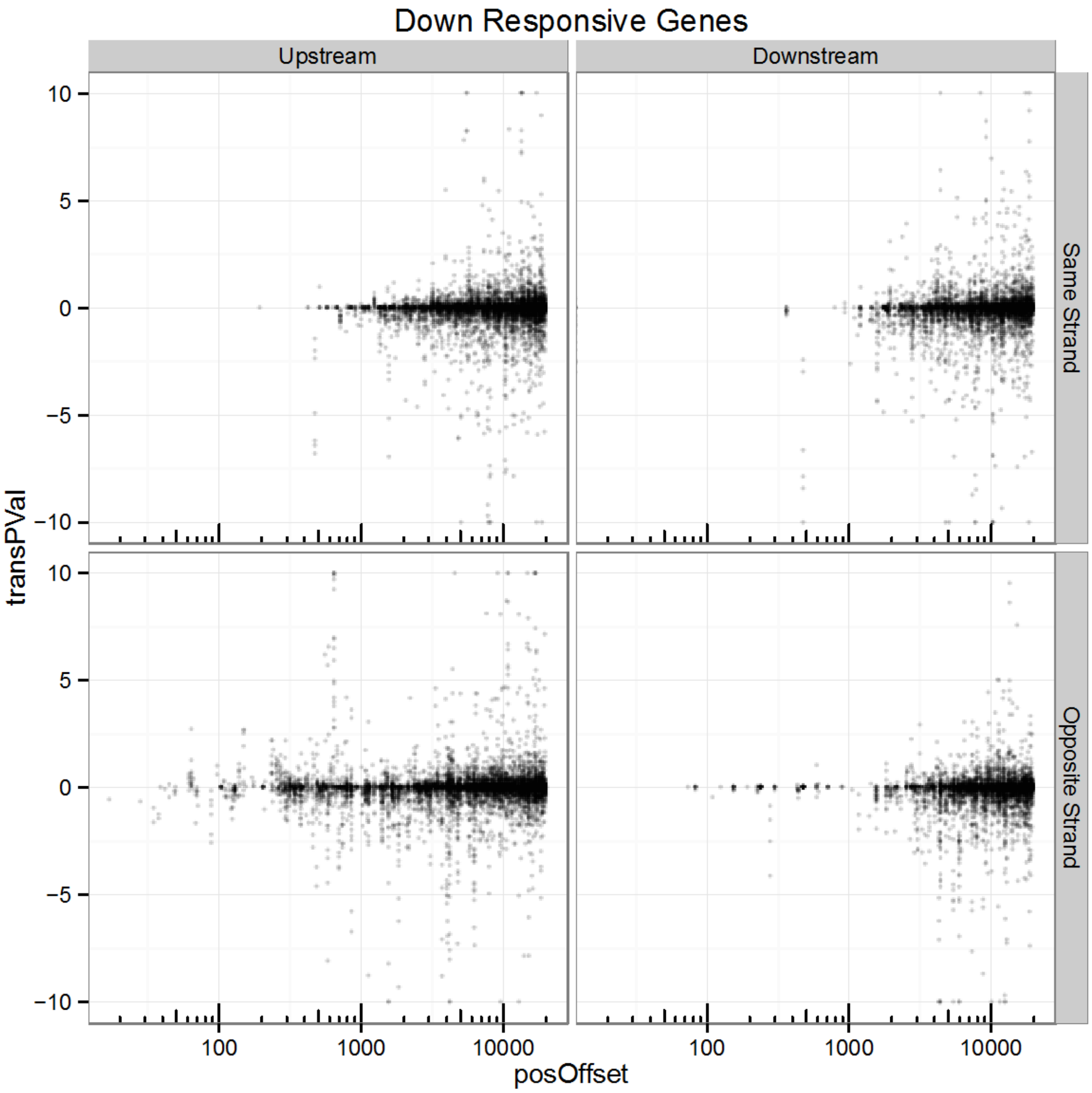

Figure S5. Raw Responsive Proximal Response. Each panel of this figure shows the raw points that produced the smoothed lines found in Figure 3. As in figure three each point represents one gene, cell lines combination for a significantly responsive gene and a proximal gene. Different panels show the responsive behavior of genes proximal to responsive genes segregated by the response direction and the promoter architecture (same/opposite strand and up/down stream promoters).
