# Supplementary material for: Diverse Hormone Response Networks in 41 Independent Drosophila Cell Lines
Source: G3 (Bethesda). 2016 Jan 12;6(3):683–94. doi: 10.1534/g3.115.023366 (PMC4777130; doi:10.1534/g3.115.023366)
Supplement: Supporting Information [file supp_g3.115.023366_TableS1.pdf]

Table S1. Additional Properties of Cell Lines in this Study

Short name	Formal name	Genotype	Additions to medium	Sex
1182-4H	1182-4H	mh^{-}	--	F(Lee *et al.* 2014)
CCa	CCa	NA	--	M (data in this study)
L1	CME L1	NA	Insulin, fly extract	M (Lee *et al.* 2014)
Cl.8	CME W1 Cl.8+	NA	Insulin, fly extract	M (Lee *et al.* 2014)
W2	CME W2	NA	Insulin, fly extract	M (Lee *et al.* 2014)
D1	D1	NA	--	M (data in this study)
DX	DX	NA	--	Ambiguous (data in this study)
E-CS	E-CS	Wild-type (Canton S)	--	F (data in this study)
E-OR	E-OR	Wild-type (Oregon R)	--	M (data in this study)
G1	G1	NA	--	M (data in this study)
G2	G2	NA	--	M (data in this study)
GM2	GM2	NA	--	M (data in this study)
GM3	GM3	NA	--	M (data in this study)
Jupiter	Jupiter	Jupiter:GFP exon trap	--	M (data in this study)
Kc	Kc167	NA	Serum-free medium CCM-3	F (Lee *et al.* 2014)
mbn2	mbn2	$mbn2^{-}$	--	M (Lee *et al.* 2014)
MCW12	MCW12	NA	Insulin, fly extract	F (data in this study)
ML83-26	ML83-26	Wild-type (Harwich)	--	F (data in this study)
BG1-c1	ML-DmBG1-c1	$y^{1}\ v^{1}\ f^{1}\ mal^{F1}$	Insulin	Ambiguous (data in this study)
BG2-c2	ML-DmBG2-c2	$y^{1}\ v^{1}\ f^{1}\ mal^{F1}$	Insulin	M (data in this study)
BG3-c2	ML-DmBG3-c2	$y^{1}\ v^{1}\ f^{1}\ mal^{F1}$	Insulin	M (Lee *et al.* 2014)
D1-c4	ML-DmD1-c4	$y^{1}\ v^{1}\ f^{1}\ mal^{F1}$	Insulin	M (data in this study)
D11	ML-DmD11	$y^{1}\ v^{1}\ f^{1}\ mal^{F1}$	Insulin	M (data in this study)
D17-c3	ML-DmD17-c3	$y^{1}\ v^{1}\ f^{1}\ mal^{F1}$	Insulin	F (Lee *et al.* 2014)
D20-c5	ML-DmD20-c5	$y^{1}\ v^{1}\ f^{1}\ mal^{F1}$	Insulin	M (Lee *et al.* 2014)
D21	ML-DmD21	$y^{1}\ v^{1}\ f^{1}\ mal^{F1}$	Insulin	M (data in this study)
D23-c4	ML-DmD23-c4	$y^{1}\ v^{1}\ f^{1}\ mal^{F1}$	Insulin	M (data in this study)
D4-c1	ML-DmD4-c1	$y^{1}\ v^{1}\ f^{1}\ mal^{F1}$	Insulin	M (Lee *et al.* 2014)
D8	ML-DmD8	$y^{1}\ v^{1}\ f^{1}\ mal^{F1}$	Insulin	F (Lee *et al.* 2014)
D9	ML-DmD9	$y^{1}\ v^{1}\ f^{1}\ mal^{F1}$	Insulin	Ambiguous (Lee *et al.* 2014)
OSS	OSS	w^{1118}; *P [*w^{+} *hsp70-bam*$^{+}$*] bam*^{D86} *ry e/bam*^{D86} *P[ovo-lacZ] P[vas-egfp]*	Insulin, fly extract, glutathione	F (data in this study)
PR-8	PR8	*Pten*^{117}; UAS-GFP; P{UAS-Ras85D^{V12}}/ {Act5C-GAL4}/	--	M (data in this study)
Pten X	Pten X	*Pten*^{117}	--	M (data in this study)
Ras-wts:RNAi	Ras[V12]; wts[RNAi]	P(Leonardi *et al.*) P{UAS-Ras85D^{V12}} P{UAS-GFP P{UAS-wts-RNAi}/ {Act5C-GAL4}	--	M (data in this study)
Ras-H3	Ras[V12]-H3	UAS-GFP; P{UAS-Ras85D^{V12}}/ {Act5C-GAL4}	--	M (data in this study)
Ras-H7	Ras[V12]-H7	UAS-GFP; P{UAS-Ras85D^{V12}}/ {Act5C-GAL4}/	--	M (data in this study)

Rumi-Ras	rumi[26] Ras[V12]-4	*rumi*^{26} P{Act5C-GAL4}/P{UAS-Ras85D^{V12}}	--	F (data in this study)
S1	S1	Wild-type (Oregon R)	--	M (LEE *et al.* 2014)
S2-DRSC	S2-DRSC	Wild-type (Oregon R)	--	M (LEE *et al.* 2014)
S3	S3	Wild-type (Oregon R)	--	M (LEE *et al.* 2014)
Sg4	Sg4	Wild-type (Oregon R)	--	M (LEE *et al.* 2014)

Listed is additional information for each cell line including genotype, variations to medium and the sex of each cell line along with the associated reference for that determination.
