# Supplementary material for: Diverse Hormone Response Networks in 41 Independent Drosophila Cell Lines
Source: G3 (Bethesda). 2016 Jan 12;6(3):683–94. doi: 10.1534/g3.115.023366 (PMC4777130; doi:10.1534/g3.115.023366)
Supplement: Supporting Information [file supp_g3.115.023366_TableS2.pdf]

Table S2. SRA Accession numbers for RNA-seq data reported in this study.

Sample	Treatment	Treatment Time	SRA Accession #
D1 C	control	----	SRR3038122
D1 E	hormone	5 h	SRR3037437
CME W1 Cl.8+ C	control	----	SRR3038123
CME W1 Cl.8+ E	hormone	5 h	SRR3037943
CME W2 C	control	----	SRR3038127
CME W2 E	hormone	5 h	SRR3037521
CME L1 C	control	----	SRR3038125
CME L1 E	hormone	5 h	SRR3038124
CCa C1	control	---- (sample 1)	SRR3038213
CCa C2	control	---- (sample 2)	SRR3038131
CCa E1	hormone	5 h (sample 1)	SRR3038201
CCa E2	hormone	5 h (sample 2)	SRR3038126
1182-4H C	control	----	SRR3038250
1182-4H E	hormone	5 h	SRR3038240
DX C	control	----	SRR3038251
DX E	hormone	5 h	SRR3038252
E-CS C	control	----	SRR3038283
E-CS E	hormone	5 h	SRR3038284
E-OR C	control	----	SRR3038285
E-OR E	hormone	5 h	SRR3038290
G1 C	control	----	SRR3038293
G1 E	hormone	5 h	SRR3038294
G2 C	control	----	SRR3038296
G2 E	hormone	5 h	SRR3038302
GM2 C	control	----	SRR3038303
GM2 E	hormone	5 h	SRR3038304
GM3 C	control	----	SRR3038306
GM3 E	hormone	5 h	SRR3040020
Jupiter C	control	----	SRR3040046
Jupiter E	hormone	5 h	SRR3040053
Kc167 C-1	control	---- (sample 1)	SRR3040054
Kc167 C-2	control	---- (sample 2)	SRR3040509
Kc167 E1-2	hormone	1 h	SRR3040556

Kc167 E3-2	hormone	3 h	SRR3040557
Kc167 E5-1	hormone	5 h (sample 1)	SRR3040058
Kc167 E5-2	hormone	5 h (sample 2)	SRR3040558
Kc167-E7-2	hormone	7 h	SRR3040559
Kc167 E24-1	hormone	24 h	SRR3040088
mbn2 C	control	----	SRR3040560
mbn2 E	hormone	5 h	SRR3040561
MCW12 C1	control	---- (sample 1)	SRR3040562
MCW12 C2	control	---- (sample 2)	SRR3040674
MCW12 E1	hormone	5 h (sample 1)	SRR3040594
MCW12 E2	hormone	5 h (sample 2)	SRR3040681
ML83-26 C	control	----	SRR3040682
ML83-26 E	hormone	5 h	SRR3041927
ML-DmBG1-c1 C	control	----	SRR3041928
ML-DmBG1-c1 E	hormone	5 h	SRR3041929
ML-DmBG2-c2 C	control	----	SRR3041930
ML-DmBG2-c2 E	hormone	5 h	SRR3041931
ML-DmBG3-c2 C-1	control	---- (sample 1)	SRR3041932
ML-DmBG3-c2 C-2	control	---- (sample 2)	SRR3041935
ML-DmBG3-c2 E1-2	hormone	1 h	SRR3041936
ML-DmBG3-c2 E3-2	hormone	3 h	SRR3041937
ML-DmBG3-c2 E5-1	hormone	5 h (sample 1)	SRR3041933
ML-DmBG3-c2 E5-2	hormone	5 h (sample 2)	SRR3041938
ML-DmBG3-c2 E7-2	hormone	7 h	SRR3041939
ML-DmBG3-c2 E24-1	hormone	24 h	SRR3041934
ML-DmD1-c4 C	control	----	SRR3041940
ML-DmD1-c4 E	hormone	5 h	SRR3041941
ML-DmD11 C	control	----	SRR3041942
ML-DmD11 E	hormone	5 h	SRR3041943
ML-DmD17-c3 C	control	----	SRR3041988
ML-DmD17-c3 E	hormone	5 h	SRR3042072
ML-DmD20-c5 C	control	----	SRR3042157
ML-DmD20-c5 E	hormone	5 h	SRR3042198
ML-DmD21 C	control	----	SRR3042199
ML-DmD21 E	hormone	5 h	SRR3042200
ML-DmD23-c4 C	control	----	SRR3042202
ML-DmD23-c4 E	hormone	5 h	SRR3042203
ML-DmD4-c1 C	control	----	SRR3042204
ML-DmD4-c1 E	hormone	5 h	SRR3042538
ML-DmD8 C	control	----	SRR3042539
ML-DmD8 E	hormone	5 h	SRR3042541
ML-DmD9 C	control	----	SRR3042543
ML-DmD9 E	hormone	5 h	SRR3042546
OSS C	control	----	SRR3042549
OSS E	hormone	5 h	SRR3042550
PR8 C	control	----	SRR3042551

PR8 E	hormone	5 h	SRR3042552
Pten X C	control	----	SRR3042553
Pten X E	hormone	5 h	SRR3042554
Ras[V12]; wts[RNAi] C	control	----	SRR3042555
Ras[V12]; wts[RNAi] E	hormone	5 h	SRR3042556
Ras[V12]-H3 C	control	----	SRR3042557
Ras[V12]-H3 E	hormone	5 h	SRR3042558
Ras[V12]-H7 C	control	----	SRR3042559
Ras[V12]-H7 E	hormone	5 h	SRR3042560
rumi[26] Ras[V12]-4 C	control	----	SRR3042561
rumi[26] Ras[V12}-4 E	hormone	5 h	SRR3042562
S1 C	control	----	SRR3042563
S1 E	hormone	5 h	SRR3042564
S2-DRSC C	control	----	SRR3042565
S2-DRSC E1	hormone	1 h	SRR3042566
S2-DRSC E3	hormone	3 h	SRR3042567
S2-DRSC E5	hormone	5 h	SRR3042568
S2 DRSC E7	hormone	7 h	SRR3042569
S2-DRSC E24	hormone	24 h	SRR3042570
S3 C	control	----	SRR3042571
S3 E	hormone	5 h	SRR3042572
Sg4 C	control	----	SRR3042573
Sg4 E	hormone	5 h	SRR3042574

The SRA accession numbers for each sample reported in this study are presented in this table.
