# Supplementary material for: Diverse Hormone Response Networks in 41 Independent Drosophila Cell Lines
Source: G3 (Bethesda). 2016 Jan 12;6(3):683–94. doi: 10.1534/g3.115.023366 (PMC4777130; doi:10.1534/g3.115.023366)
Supplement: Supporting Information [file supp_g3.115.023366_TableS8.pdf]

Table S8. Robustness of RGC to Thresholds.

		Fold Change Threshold						
		**log2(6/5)**	**log2(3/2)**	**log2(5/3)**	**log2(2)**	**log2(5/2)**	**log2(3)**	**log2(4)**
**P-Value Threshold**	**0.001**	0.9862	0.9862	0.9862	0.9889	0.9938	0.9900	0.9714
	**0.0025**	0.9916	0.9916	0.9916	0.9954	0.9954	0.9898	0.9684
	**0.005**	0.9938	0.9938	0.9938	0.9985	0.9955	0.9885	0.9665
	**0.01**	0.9950	0.9950	0.9960	1.0000	0.9949	0.9875	0.9659
	**0.025**	0.9929	0.9929	0.9966	0.9978	0.9922	0.9862	0.9633
	**0.05**	0.9915	0.9934	0.9960	0.9949	0.9890	0.9846	0.9633
	**0.1**	0.9899	0.9923	0.9913	0.9841	0.9834	0.9809	0.9629

For a range of fold change and p-value thresholds the correlation of the RGC with the RGC calculated using the chosen threshold values are shown. Cell values are the Pearson Correlation between the used thresholds and other thresholds on the number of identified hits.
