# Supplementary material for: Diverse Hormone Response Networks in 41 Independent Drosophila Cell Lines
Source: G3 (Bethesda). 2016 Jan 12;6(3):683–94. doi: 10.1534/g3.115.023366 (PMC4777130; doi:10.1534/g3.115.023366)
Supplement: Supporting Information [file supp_g3.115.023366_TableS10.pdf]

Table S10. Overlap with Previous Ecdysone Response Studies.

Response	Genes	Paper	Tissue /Cell Line	Early/Late	Sequencing Tech.	P-Value	% Hits	# Overlap	# Hits	# Other Hits
Induced	All	Gauhar et al. 2009	Kc	Ealry (1, 3,6)	Microarray	6.86E-82	9.34	87	931	112
Induced	All	Gauhar et al. 2009	Kc	Late (12, 24, 48)	Microarray	2.64E-62	12.03	112	931	267
Induced	All	Gonsalves et al. 2011	Kc	Ealry (4)	Microarray	1.97E-12	2.58	24	931	71
Induced	All	Gonsalves et al. 2011	Salivary	Ealry (4)	Microarray	1.93E-15	3.65	34	931	109
Induced	All	Beckstead et al. 2005	W3 Larvae	Early (6)	Microarray	2.81E-69	16.86	157	931	478
Induced	All	Shlyueva et al. 2014	S2	Late (24)	RNA-seq	4.33E-107	32.22	300	931	1159
Induced	Widespread	Gauhar et al. 2009	Kc	Ealry (1, 3,6)	Microarray	5.04E-44	41.18	28	68	112
Induced	Widespread	Gauhar et al. 2009	Kc	Late (12, 24, 48)	Microarray	7.24E-34	42.65	29	68	267
Induced	Widespread	Gonsalves et al. 2011	Kc	Ealry (4)	Microarray	1.55E-12	13.24	9	68	71
Induced	Widespread	Gonsalves et al. 2011	Salivary	Ealry (4)	Microarray	2.88E-09	11.76	8	68	109
Induced	Widespread	Beckstead et al. 2005	W3 Larvae	Early (6)	Microarray	1.39E-27	44.12	30	68	478
Induced	Widespread	Shlyueva et al. 2014	S2	Late (24)	RNA-seq	5.41E-38	70.59	48	68	1159
Induced	Restricted	Gauhar et al. 2009	Kc	Ealry (1, 3,6)	Microarray	1.14E-42	6.84	59	863	112
Induced	Restricted	Gauhar et al. 2009	Kc	Late (12, 24, 48)	Microarray	3.18E-37	9.62	83	863	267
Induced	Restricted	Gonsalves et al. 2011	Kc	Ealry (4)	Microarray	5.94E-06	1.74	15	863	71
Induced	Restricted	Gonsalves et al. 2011	Salivary	Ealry (4)	Microarray	4.30E-10	3.01	26	863	109
Induced	Restricted	Beckstead et al. 2005	W3 Larvae	Early (6)	Microarray	4.77E-48	14.72	127	863	478
Induced	Restricted	Shlyueva et al. 2014	S2	Late (24)	RNA-seq	3.05E-78	29.20	252	863	1159
Repressed	All	Gauhar et al. 2009	Kc	Ealry (1, 3,6)	Microarray	4.62E-17	3.14	25	797	60
Repressed	All	Gauhar et al. 2009	Kc	Late (12, 24, 48)	Microarray	1.43E-15	9.66	77	797	510
Repressed	All	Gonsalves et al. 2011	Kc	Ealry (4)	Microarray	3.18E-09	2.38	19	797	73
Repressed	All	Beckstead et al. 2005	W3 Larvae	Early (6)	Microarray	0.000236953	1.76	14	797	92
Repressed	All	Shlyueva et al. 2014	S2 Late	Late (24)	RNA-seq	5.86E-57	28.86	230	797	1347
Repressed	Widespread	Gauhar et al. 2009	Kc	Ealry (1, 3,6)	Microarray	6.35E-11	18.75	6	32	60
Repressed	Widespread	Gauhar et al. 2009	Kc	Late (12, 24, 48)	Microarray	1.58E-06	25.00	8	32	510
Repressed	Widespread	Gonsalves et al. 2011	Kc	Ealry (4)	Microarray	2.32E-05	9.38	3	32	73
Repressed	Widespread	Beckstead et al. 2005	W3 Larvae	Early (6)	Microarray	0.019172858	3.12	1	32	92
Repressed	Widespread	Shlyueva et al. 2014	S2 Late	Late (24)	RNA-seq	8.26E-10	50.00	16	32	1347

Repressed	Restricted	Gauhar et al. 2009	Kc	Ealry (1, 3,6)	Microarray	3.04E-11	2.48	19	765	60
Repressed	Restricted	Gauhar et al. 2009	Kc	Late (12, 24, 48)	Microarray	1.32E-12	9.02	69	765	510
Repressed	Restricted	Gonsalves et al. 2011	Kc	Ealry (4)	Microarray	3.52E-07	2.09	16	765	73
Repressed	Restricted	Beckstead et al. 2005	W3 Larvae	Early (6)	Microarray	0.000517665	1.7	13	765	92
Repressed	Restricted	Shlyueva et al. 2014	S2 Late	Late (24)	RNA-seq	2.42E-50	27.97	214	765	1347

The overlap of widespread, restricted and all responsive genes with previous studies are shown along with the hypergeometric p-value for this overlap.
