# Supplementary material for: Diverse Hormone Response Networks in 41 Independent Drosophila Cell Lines
Source: G3 (Bethesda). 2016 Jan 12;6(3):683–94. doi: 10.1534/g3.115.023366 (PMC4777130; doi:10.1534/g3.115.023366)
Supplement: Supporting Information [file supp_g3.115.023366_TableS11.pdf]

Table S11. Divergently Responsive Genes.

Gene Name	Number of Induced Cell Lines	Number of Repressed Cell Lines
Oatp74D	5	16
CG9932	5	6
alpha-Est9	10	4
CG13252	4	10
Mal-A5	4	3
sda	3	9
SKIP	3	9
Mct1	9	2
CG32425	6	2
Toll-7	4	2
CG9743	2	21
Argk	2	8
Timp	2	7
CG3036	2	6
bmm	2	5
Pino	2	3
Ama	36	1
CG11509	26	1
CG9416	26	1
br	23	1
eater	20	1
Cyp12a4	13	1
Ugt58Fa	7	1
CG17278	4	1
CrebA	4	1
Apoltp	3	1
bnl	3	1
Cyp9f2	2	1
Dyrk2	2	1
kon	2	1
sick	2	1

Swim	2	1
Cyp6a13	1	17
CG31710	1	14
CG7255	1	14
GstT4	1	14
Fas3	1	13
Ndae1	1	13
babos	1	12
yin	1	11
CG15695	1	10
CG31522	1	10
Lrch	1	10
CG17646	1	9
rau	1	9
CG43795	1	7
Rcd2	1	7
CG3376	1	6
Ih	1	6
CG6357	1	5
CG9521	1	5
pgant2	1	5
CG10527	1	4
CG14995	1	4
CG18446	1	4
nrv3	1	4
ths	1	4
CG42240	1	3
Drat	1	3
Imp	1	3
tutl	1	3
CBP	1	2
CG31219	1	2
CG9981	1	2
PH4alphaEFB	1	2
Prestin	1	2
btl	1	1
CG13868	1	1
CG17124	1	1
CG17150	1	1
CG17834	1	1
CG34232	1	1
CG43164	1	1
CR44526	1	1
Cyp6w1	1	1
E2f	1	1
ena	1	1
Idgf3	1	1
Mbs	1	1

Plod	1	1
stai	1	1
sty	1	1
Traf4	1	1

This table shows the genes that respond significantly inductively in some cell line(s) and significantly repressively in other cell line(s).
