# Supplementary material for: Diverse Hormone Response Networks in 41 Independent Drosophila Cell Lines
Source: G3 (Bethesda). 2016 Jan 12;6(3):683–94. doi: 10.1534/g3.115.023366 (PMC4777130; doi:10.1534/g3.115.023366)
Supplement: Supporting Information [file supp_g3.115.023366_TableS13.pdf]

Table S13. Most Correlated Genes with Fraction of *EcR-B1/2* Isoform.

Gene	Correlation with Fraction of Short EcR Isofrom	Permutation Test P-Value	Adjusted P-Value
glec	-0.644928268	0	0
sqz	0.660407469	4.00E-06	0.012388333
CG5059	0.59833374	8.00E-06	0.012388333
Eip55E	0.571066986	8.00E-06	0.012388333
br	0.633163455	1.20E-05	0.012388333
CG5335	0.634343237	1.40E-05	0.012388333
CG14440	0.599282244	1.40E-05	0.012388333
Xbp1	-0.586784569	1.50E-05	0.012388333
CG4825	0.560477019	1.50E-05	0.012388333
CG5482	0.581179456	2.00E-05	0.014294231
mlt	0.483261053	2.30E-05	0.014294231
LIMK1	0.602181835	2.50E-05	0.014294231
cdi	-0.617214213	2.50E-05	0.014294231
CG34330	-0.581866181	3.50E-05	0.0185825
CG5359	0.5953757	4.00E-05	0.019238353
Alh	0.577959631	4.30E-05	0.019238353
loj	-0.57636296	4.40E-05	0.019238353
CG2865	0.496826963	5.70E-05	0.022690211
osp	-0.574710543	5.80E-05	0.022690211
Tsp42Ee	-0.598284087	6.30E-05	0.02341395
CG31370	0.576136587	8.40E-05	0.029219379
CG1418	-0.550475603	9.10E-05	0.029219379
tws	0.495246729	9.10E-05	0.029219379
Glycogenin	0.55734144	9.90E-05	0.029219379
Syp	0.54993558	0.00011	0.029219379
CG32428	0.594831217	0.000111	0.029219379
CG3408	0.551354562	0.000113	0.029219379
SdhA	-0.529640291	0.000114	0.029219379
Klp64D	-0.528184046	0.000114	0.029219379

CG2247	0.534616716	0.000122	0.030227533
CG9005	0.544531433	0.00014	0.033568387
cnc	0.526806645	0.000147	0.033885735
Hmg-2	-0.534014497	0.000151	0.033885735
Rel	-0.542420057	0.000155	0.033885735
CG13624	0.556401565	0.000174	0.036952629
Cbp80	0.499257521	0.000196	0.039575703
CG3587	0.539226318	0.000197	0.039575703
CycE	0.53696766	0.000206	0.040173595
Sans	-0.478439908	0.000218	0.040173595
RabX1	0.559355235	0.000221	0.040173595
mnb	0.50065999	0.000222	0.040173595
Pbgs	-0.51176193	0.000227	0.040173595
Su(dx)	-0.497795752	0.000251	0.043387977
CG9323	-0.541485883	0.000276	0.046625182
Itgbetanu	0.520438966	0.000288	0.0475712
CG11739	-0.529675673	0.000314	0.050133213
CG17036	0.504040971	0.000317	0.050133213
CG9149	-0.504389999	0.00033	0.050719294
SCOT	-0.486500621	0.000342	0.050719294
ihog	0.51369445	0.000347	0.050719294
CG14971	-0.520464111	0.000348	0.050719294
ena	0.493287985	0.000377	0.052534932
tai	0.472951129	0.000382	0.052534932
CG3781	0.503481214	0.00039	0.052534932
CG43658	0.467565063	0.000397	0.052534932
E2f	0.502944659	0.000404	0.052534932
rho	-0.521886478	0.000406	0.052534932
CG12560	0.494852351	0.000415	0.052534932
Cog7	-0.505347582	0.000417	0.052534932
CG7943	-0.493436474	0.000453	0.05611915
CG8858	0.473405421	0.000497	0.060063435
jet	-0.478195889	0.000501	0.060063435
CG17029	0.474493467	0.000554	0.063968848
rhea	0.496542017	0.000562	0.063968848
peb	0.498685766	0.000564	0.063968848
qkr58E-1	0.476232951	0.000568	0.063968848
CG15170	0.471215173	0.000577	0.064012552
CG14906	-0.485501257	0.000598	0.065366676
CG9743	-0.51104703	0.000611	0.065819754
CG7484	-0.488968967	0.000657	0.068924182
Lasp	0.488775298	0.000674	0.068924182
Tapdelta	-0.478834328	0.000682	0.068924182
CG9590	-0.472750658	0.000691	0.068924182
CG6330	-0.469422457	0.000699	0.068924182
RnrS	0.485264857	0.000701	0.068924182
CG9705	0.49725942	0.00071	0.068924182
CG7556	-0.496916291	0.000714	0.068924182

CG3703	0.502312479	0.000732	0.069155127
GlcAT-P	-0.510481353	0.000735	0.069155127
CG7565	-0.469127833	0.000756	0.07024185
Prestin	0.488607675	0.000823	0.075522951
CG31777	-0.485442491	0.000883	0.079524145
garz	-0.49225042	0.000888	0.079524145
CG14907	-0.476211731	0.000922	0.081586024
dmGlut	0.487515735	0.000977	0.085435776
mdlc	0.485664353	0.001023	0.088418128
Sec24CD	-0.481288453	0.001051	0.089794057
CG5001	-0.477730496	0.001098	0.092286124
CG32262	-0.467627802	0.001105	0.092286124
Mal-B2	-0.497905227	0.00113	0.093325444
Cyp28d1	-0.475526108	0.00128	0.104552088
CG15111	-0.468546288	0.001313	0.106081837
CG7265	0.47074843	0.001413	0.112933645
CG6479	0.467233347	0.001455	0.115053351
Letm1	-0.472584042	0.001494	0.116893705
dre4	0.476710975	0.001587	0.122876781
RhoL	-0.472579311	0.001747	0.132959684
CG34376	0.497281292	0.001753	0.132959684
bur	0.471887383	0.001822	0.136797232
CG31098	0.472187578	0.002067	0.15364011

The genes with normalized expression showing the largest correlation with the fraction of the *ErC-B1/2* isoform are shown in this table.
