# Supplementary material for: Diverse Hormone Response Networks in 41 Independent Drosophila Cell Lines
Source: G3 (Bethesda). 2016 Jan 12;6(3):683–94. doi: 10.1534/g3.115.023366 (PMC4777130; doi:10.1534/g3.115.023366)
Supplement: Supporting Information [file supp_g3.115.023366_FigureS1.pdf]

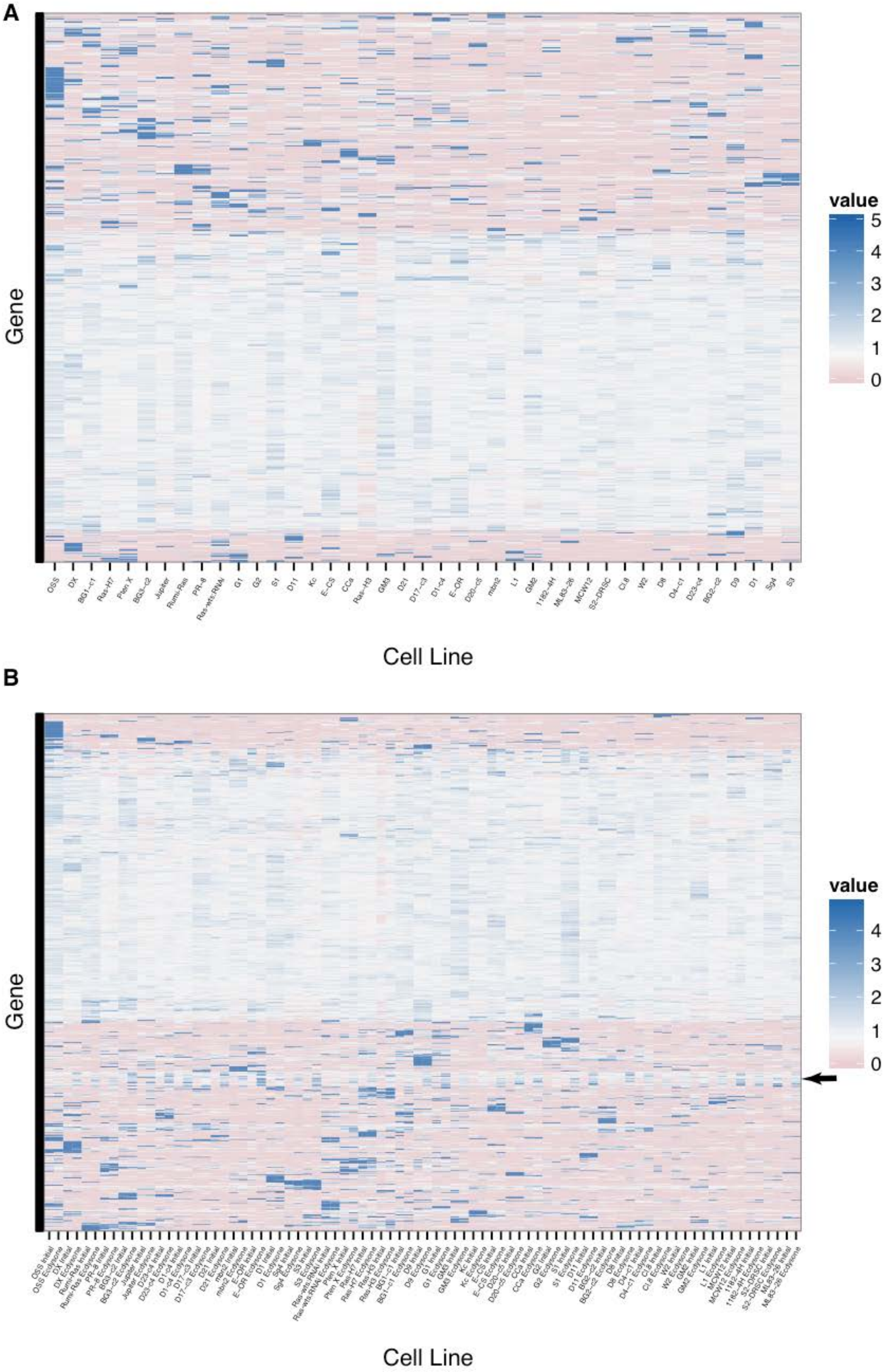
A
Gene
value
5
4
3
2
1
0
OSS
DX
BG1–c1
Ras-H7
Pten X
BG3–c2
Jupiter
Rumi-Ras
PR–8
Ras-wts-RNAi
G1
G2
S1
D11
Kc
E–CS
CCa
Ras–H3
GM3
D21
D17–c3
D1-c4
E–OR
D20–c5
mbn2
L1
GM2
1182–4H
ML83–26
MCW12
S2–DRSC
Cl.8
W2
D8
D4–c1
D23–c4
BG2–c2
D9
D1
Sg4
S3
Cell Line
B
Gene
value
4
3
2
1
0
Cell Line

Figure S1. Transcription Factor Expression Diversity. This figure is a heat map representing the expression levels of all transcription factors at the A) zero hour time point and B) the zero and five hour exposure time point. The expression levels are normalized by the mean of expression across all cell lines. Both genes and cell lines are hierarchically clustered based on Euclidian distance. The arrow points the cluster of ecdysone responsive genes as noted by the striped pattern of alternative low and high expression.
