# Supplementary material for: Diverse Hormone Response Networks in 41 Independent Drosophila Cell Lines
Source: G3 (Bethesda). 2016 Jan 12;6(3):683–94. doi: 10.1534/g3.115.023366 (PMC4777130; doi:10.1534/g3.115.023366)
Supplement: Supporting Information [file supp_g3.115.023366_TableS14.pdf]

Table S14. Most Important Covariates.

Gene	Correlation with Fraction of Short EcR Isofrom	Permutation Test P-Value	Adjusted P-Value
glec	-0.644928268	0	0
sqz	0.660407469	4.00E-06	0.012388333
CG5059	0.59833374	8.00E-06	0.012388333
Eip55E	0.571066986	8.00E-06	0.012388333
br	0.633163455	1.20E-05	0.012388333
CG5335	0.634343237	1.40E-05	0.012388333
CG14440	0.599282244	1.40E-05	0.012388333
Xbp1	-0.586784569	1.50E-05	0.012388333
CG4825	0.560477019	1.50E-05	0.012388333
CG5482	0.581179456	2.00E-05	0.014294231
mlt	0.483261053	2.30E-05	0.014294231
LIMK1	0.602181835	2.50E-05	0.014294231
cdi	-0.617214213	2.50E-05	0.014294231
CG34330	-0.581866181	3.50E-05	0.0185825
CG5359	0.5953757	4.00E-05	0.019238353
Alh	0.577959631	4.30E-05	0.019238353

loj	-0.57636296	4.40E-05	0.019238353
CG2865	0.496826963	5.70E-05	0.022690211
osp	-0.574710543	5.80E-05	0.022690211
Tsp42Ee	-0.598284087	6.30E-05	0.02341395
CG31370	0.576136587	8.40E-05	0.029219379
CG1418	-0.550475603	9.10E-05	0.029219379
tws	0.495246729	9.10E-05	0.029219379
Glycogenin	0.55734144	9.90E-05	0.029219379
Syp	0.54993558	0.00011	0.029219379
CG32428	0.594831217	0.000111	0.029219379
CG3408	0.551354562	0.000113	0.029219379
SdhA	-0.529640291	0.000114	0.029219379
Klp64D	-0.528184046	0.000114	0.029219379
CG2247	0.534616716	0.000122	0.030227533
CG9005	0.544531433	0.00014	0.033568387
cnc	0.526806645	0.000147	0.033885735
Hmg-2	-0.534014497	0.000151	0.033885735
Rel	-0.542420057	0.000155	0.033885735
CG13624	0.556401565	0.000174	0.036952629
Cbp80	0.499257521	0.000196	0.039575703
CG3587	0.539226318	0.000197	0.039575703
CycE	0.53696766	0.000206	0.040173595
Sans	-0.478439908	0.000218	0.040173595
RabX1	0.559355235	0.000221	0.040173595
mnb	0.50065999	0.000222	0.040173595
Pbgs	-0.51176193	0.000227	0.040173595
Su(dx)	-0.497795752	0.000251	0.043387977
CG9323	-0.541485883	0.000276	0.046625182
Itgbetanu	0.520438966	0.000288	0.0475712
CG11739	-0.529675673	0.000314	0.050133213
CG17036	0.504040971	0.000317	0.050133213
CG9149	-0.504389999	0.00033	0.050719294
SCOT	-0.486500621	0.000342	0.050719294
ihog	0.51369445	0.000347	0.050719294
CG14971	-0.520464111	0.000348	0.050719294
ena	0.493287985	0.000377	0.052534932
tai	0.472951129	0.000382	0.052534932
CG3781	0.503481214	0.00039	0.052534932
CG43658	0.467565063	0.000397	0.052534932
E2f	0.502944659	0.000404	0.052534932
rho	-0.521886478	0.000406	0.052534932
CG12560	0.494852351	0.000415	0.052534932
Cog7	-0.505347582	0.000417	0.052534932
CG7943	-0.493436474	0.000453	0.05611915
CG8858	0.473405421	0.000497	0.060063435
jet	-0.478195889	0.000501	0.060063435
CG17029	0.474493467	0.000554	0.063968848
rhea	0.496542017	0.000562	0.063968848

peb	0.498685766	0.000564	0.063968848
qkr58E-1	0.476232951	0.000568	0.063968848
CG15170	0.471215173	0.000577	0.064012552
CG14906	-0.485501257	0.000598	0.065366676
CG9743	-0.51104703	0.000611	0.065819754
CG7484	-0.488968967	0.000657	0.068924182
Lasp	0.488775298	0.000674	0.068924182
Tapdelta	-0.478834328	0.000682	0.068924182
CG9590	-0.472750658	0.000691	0.068924182
CG6330	-0.469422457	0.000699	0.068924182
RnrS	0.485264857	0.000701	0.068924182
CG9705	0.49725942	0.00071	0.068924182
CG7556	-0.496916291	0.000714	0.068924182
CG3703	0.502312479	0.000732	0.069155127
GlcAT-P	-0.510481353	0.000735	0.069155127
CG7565	-0.469127833	0.000756	0.07024185
Prestin	0.488607675	0.000823	0.075522951
CG31777	-0.485442491	0.000883	0.079524145
garz	-0.49225042	0.000888	0.079524145
CG14907	-0.476211731	0.000922	0.081586024
dmGlut	0.487515735	0.000977	0.085435776
mdlc	0.485664353	0.001023	0.088418128
Sec24CD	-0.481288453	0.001051	0.089794057
CG5001	-0.477730496	0.001098	0.092286124
CG32262	-0.467627802	0.001105	0.092286124
Mal-B2	-0.497905227	0.00113	0.093325444
Cyp28d1	-0.475526108	0.00128	0.104552088
CG15111	-0.468546288	0.001313	0.106081837
CG7265	0.47074843	0.001413	0.112933645
CG6479	0.467233347	0.001455	0.115053351
Letm1	-0.472584042	0.001494	0.116893705
dre4	0.476710975	0.001587	0.122876781
RhoL	-0.472579311	0.001747	0.132959684
CG34376	0.497281292	0.001753	0.132959684
bur	0.471887383	0.001822	0.136797232
CG31098	0.472187578	0.002067	0.15364011

This table includes the transcription factor importance values as well as importance values relative to the 20th most important variable from the model fitting procedure for the top most important variables.
