# Supplementary material for: Diverse Hormone Response Networks in 41 Independent Drosophila Cell Lines
Source: G3 (Bethesda). 2016 Jan 12;6(3):683–94. doi: 10.1534/g3.115.023366 (PMC4777130; doi:10.1534/g3.115.023366)
Supplement: Supporting Information [file supp_g3.115.023366_FigureS2.pdf]

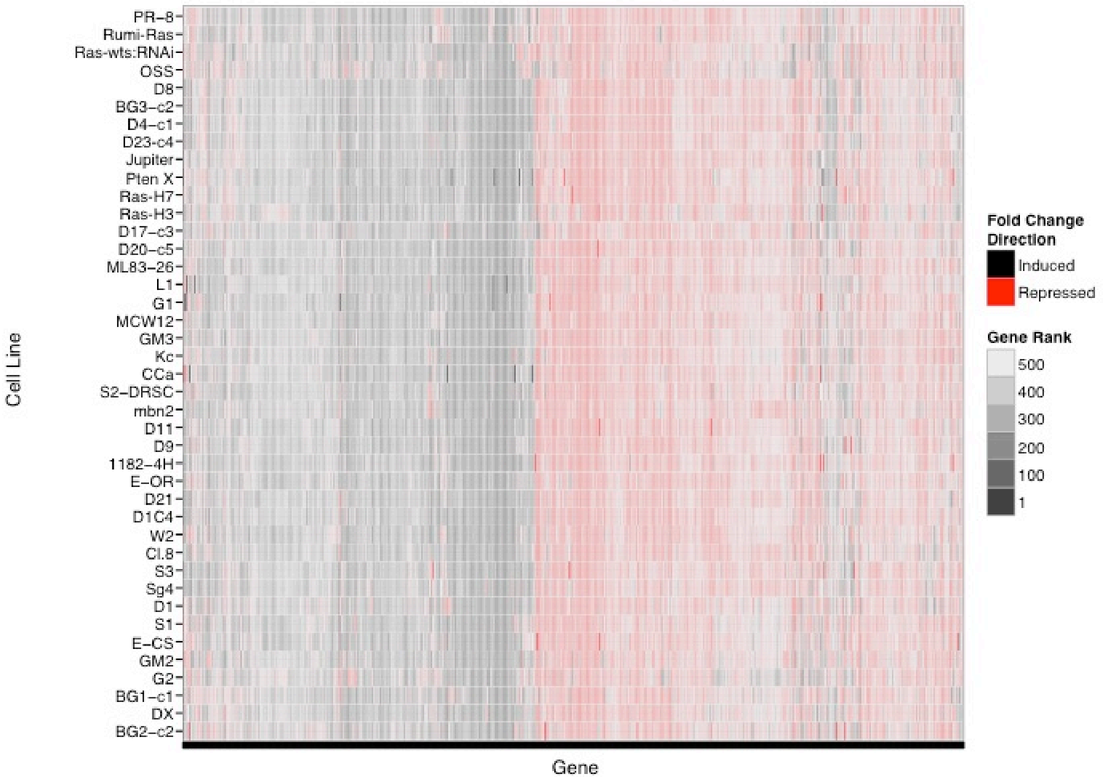

Figure S2. Gene Level Clustering and Similarity. This figure is a heat map representing the response of all genes (along the x axis) that respond significantly in at least two cell lines. The color of each cell represents the direction of response (log fold change greater or less than zero) and the level of transparency represents the rank of the log fold change within each cell line. Both cell lines and genes are clustered (using a hierarchical clustering algorithm).
