# Supplementary material for: Diverse Hormone Response Networks in 41 Independent Drosophila Cell Lines
Source: G3 (Bethesda). 2016 Jan 12;6(3):683–94. doi: 10.1534/g3.115.023366 (PMC4777130; doi:10.1534/g3.115.023366)
Supplement: Supporting Information [file supp_g3.115.023366_FigureS3.pdf]

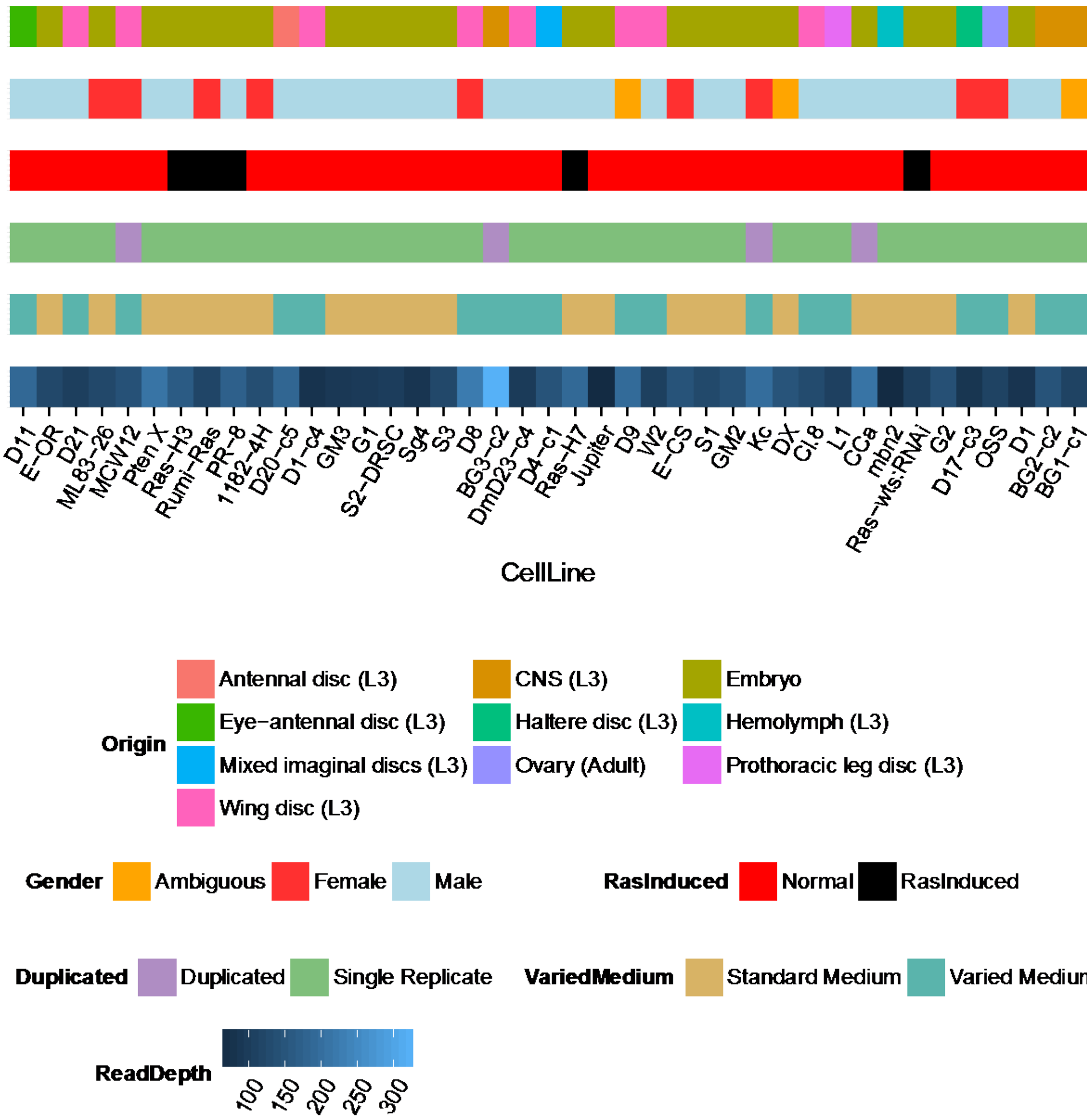

Figure S3. Cell Line Characteristics Clustered by Restricted Ecdysone Response. The cell lines in this figure are clustered in the same order as in Figure 1. Shown in the six panels are the tissue of origin, the gender, the inclusion of constitutively active Ras85D, whether the cell line was measured in biological duplicate, whether a standard media was used and the read depth for all samples from each cell line. The clustering of these covariates with the restricted response is discussed in the main text.
